# Supraphysiological Levels of Oxygen Exposure During the Neonatal Period Impairs Signaling Pathways Required for Learning and Memory

**DOI:** 10.1038/s41598-018-28220-4

**Published:** 2018-07-02

**Authors:** Manimaran Ramani, Ranjit Kumar, Brian Halloran, Charitharth Vivek Lal, Namasivayam Ambalavanan, Lori L. McMahon

**Affiliations:** 1 Departments of Pediatrics, University of Alabama at Birmingham, Birmingham, AL 35233 USA; 2 Departments of Bioinformatics, University of Alabama at Birmingham, Birmingham, AL 35233 USA; 3 Departments of Cell, Developmental, and Integrative Biology, University of Alabama at Birmingham, Birmingham, AL 35233 USA; 4 Departments of Neurobiology, University of Alabama at Birmingham, Birmingham, AL 35233 USA

## Abstract

Preterm infants often require prolonged oxygen supplementation and are at high risk of neurodevelopmental impairment. We recently reported that adult mice exposed to neonatal hyperoxia (postnatal day [P] 2 to 14) had spatial navigation memory deficits associated with hippocampal shrinkage. The mechanisms by which early oxidative stress impair neurodevelopment are not known. Our objective was to identify early hyperoxia-induced alterations in hippocampal receptors and signaling pathways necessary for memory formation. We evaluated C57BL/6 mouse pups at P14, exposed to either 85% oxygen or air from P2 to 14. We performed targeted analysis of hippocampal ligand-gated ion channels and proteins necessary for memory formation, and global bioinformatic analysis of differentially expressed hippocampal genes and proteins. Hyperoxia decreased hippocampal mGLU7, TrkB, AKT, ERK2, mTORC1, RPS6, and EIF4E and increased α3, α5, and ɤ2 subunits of GABA_A_ receptor and PTEN proteins, although changes in gene expression were not always concordant. Bioinformatic analysis indicated dysfunction in mitochondria and global protein synthesis and translational processes. In conclusion, supraphysiological oxygen exposure reduced proteins necessary for hippocampus-dependent memory formation and may adversely impact hippocampal mitochondrial function and global protein synthesis. These early hippocampal changes may account for memory deficits seen in preterm survivors following prolonged oxygen supplementation.

## Introduction

Children born preterm with a relatively uncomplicated neonatal intensive care unit course often have deficits in executive function and learning and memory^1–3^. Compared to adolescents born at term, adolescents born preterm have reduced hippocampal volume that is associated with long-term memory deficits^4^. *In utero*, the brain normally develops in a relatively hypoxemic environment (PaO_2_ < 50 mm Hg) although oxygen delivery is usually adequate^5–7^. In contrast, brain development in preterm infants occurs in a relatively hyperoxemic environment (PaO_2_ > 50 mm Hg)^8^ compared to fetuses *in utero* at the same gestational age even when their oxygen saturation is maintained within a narrow target range^9^, and more extreme hyperoxemia may occur when preterm infants are on supplemental oxygen. Preterm infants requiring prolonged periods of supplemental oxygen are at higher risk of morbidities of prematurity such as retinopathy of prematurity^10,11^, chronic lung disease (bronchopulmonary dysplasia [BPD])^12,13^, and deficits in executive function and cognition even in the absence of intraventricular hemorrhage^14–17^.

Brain development in newborn mouse pups corresponds to 24–28 weeks of gestation in human preterm infants^18^. Recently, we have shown that oxygen exposure during a critical developmental period (P2–14) impaired spatial navigation and memory performance, reduced anxiety and fear, and hippocampal atrophy when assessed later in young adult life (14 weeks)^19^. We have also shown that administration of retinoids to newborn mice pups during hyperoxia exposure mitigate oxygen-induced spatial navigation memory deficits and hippocampal atrophy^20^. Oxygen exposure is known to cause neuronal cell death, reduction in neurotrophin mRNA expression, and inactivation of neuronal survival signaling proteins^21,22^. While most of the regions of the brain tolerate oxidative stress well, neurons in the CA1 region of the hippocampus are particularly vulnerable to oxidative stress^23^. Mitochondria isolated from hippocampal CA1 neurons release more reactive oxygen species than those from CA3^24^. Neurons in the CA1 region are particularly vulnerable to oxidative stress induced calcium dysregulation^25^ and glutamate hyperactivity^26^. However, the mechanisms underlying oxygen toxicity on the neurodevelopment and long-term function needs to be further defined.

The hippocampus plays a critical role in the formation and retrieval of memories. Activation of N-Methyl-D-aspartic acid (NMDA) receptors, α-amino-3-hydroxy-5-methyl-4-isoxazolepropionic acid (AMPA) receptors, calmodulin, and tropomyosin receptor kinase B (TrkB)/extracellular signal-regulated kinase (ERK)/ phosphoinositide 3-kinase (PI3K) signaling pathways are necessary for memory formation^27–29^. We hypothesized that prolonged supraphysiological levels of oxygen exposure in the developing hippocampus would impair these pathways necessary for memory formation and storage. Our objective was therefore to determine the changes in the hippocampal ligand-gated ion channels and signaling pathways necessary for long-term memory formation in newborn mice exposed to hyperoxia during critical brain developmental period. We chose a timepoint immediately following hyperoxia exposure (P14) to evaluate mice, for we anticipated that evaluation of transcriptomic and proteomic data from later time points would indicate results of hyperoxia-induced hippocampal shrinkage (e.g. signals due to fewer neurons but more glia), but would not indicate the mechanisms contributing to such oxidative injury.

## Materials and Methods

All protocols were approved by the UAB Institutional Animal Care and Use Committee (IACUC) and were consistent with the PHS Policy on Humane Care and Use of Laboratory Animals (Office of Laboratory Animal Welfare, Aug 2002) and the Guide for the Care and Use of Laboratory Animals (National Research Council, National Academy Press, 1996).

### Animal model

C57BL/6 dams and their pups of both sexes were exposed to either normobaric hyperoxia (85% O_2_, N = 6) or normobaric 21% O_2_ ambient air (Air, N = 5) from the second postnatal day (P2) until postnatal day 14 (P14) as described earlier^20^. An additional set of mice that were exposed to 85% O_2_ (Hyperoxia, N = 6) or 21% O_2_ (Air, N = 6) were returned to room air and maintained on standard rodent diet and light/dark cycling in microisolator cages until immunoblotting assessment at 14 weeks of age.

At P14, hippocampal proteins were analyzed by unbiased proteomic profiling using mass spectroscopy. Initially, the targeted analysis was performed for ligand-gated ion channels (glutamate [NMDA and AMPA] and gamma-aminobutyric acid [GABA] receptors) and proteins, synaptic SNARE molecules, and transcription factors required for normal hippocampus-dependent learning and memory. Subsequently, bioinformatics analysis was performed on other differentially expressed hippocampal proteins between hyperoxia and air-exposed groups. In addition, targeted gene expression analysis was also performed for the ligand-gated ion channels, synaptic SNARE molecules, and transcription factors required for normal hippocampus-dependent learning and memory. To determine changes in the level of proteins of interest (total TrkB, total AKT, and total ERK2) over time, immunoblotting was performed from P14 and 14 week old mouse hippocampal samples.

Assays for 8-hydroxy-2′-deoxyguanosine (8OHdG: oxidative DNA damage marker) and malondialdehyde adducts (MDA: lipid peroxidation marker) were done on P14 hippocampal homogenates. We performed immunohistochemical analysis at P14 in an additional set of mice exposed to 85% O2 (Hyperoxia, N = 6) or 21% O2 (Air, N = 6) to (a) determine alterations in the rate of cell proliferation in the dentate gyrus (proliferative cell nuclear antigen [PCNA]). (b), CA1 region reactive astrocytes (glial fibrillary acidic protein [GFAP]), and (c) myelin protein (2′, 3′-Cyclic-nucleotide 3′-phosphodiesterase, [CNPase])

### Protein Isolation

Following cervical dislocation, the whole brain was harvested, and hippocampi were removed in a sterile manner. Upon harvesting, the right hippocampus was flash frozen in a steel beaded Eppendorf tube. Tissue was then homogenized using Qiagen tissue lyser (Qiagen, MD, USA) and T-PER + Halt protease inhibitors + PMSF solution and protein assay was performed using BCA protein assay kit (Thermo Fisher Scientific, MA, USA)^30^. The left hippocampus was also flash frozen in separate steel beaded Eppendorf tube for mRNA isolation.

### Mass Spectrometry

The mass spectrometric analysis of hippocampal proteins was done as previously described^31^. Briefly, using a split flow configuration, peptide digests were injected into a dSurveyor HPLC plus (Thermo Fisher Scientific, MA, USA) and data were collected in CID mode. The fragmentation scans were obtained at a 60 K resolution with a minimum signal threshold of 2000 counts. For the dependent scans, charge state screening was enabled, and the dynamic exclusion was enabled with the following settings: repeat count 2, repeat duration 15 s, exclusion list size 500, and exclusion duration 60 s.

The XCalibur RAW files were collected in profile mode, centroided, and later converted to MzXML using ReAdW v. 3.5.1. The mgf files were then created using MzXML2Search (included in TPP v. 3.5) for all scans with a precursor mass between 350 and 1200 Da. The data were then searched using SEQUEST (a tandem mass spectrometry data analysis program) which was set for three maximum missed cleavages, a precursor mass window of 20 ppm, trypsin digestion, variable modification C at 57.0293, and M at 15.9949.

A list of peptide IDs were generated based on SEQUEST search results, which were filtered using Scaffold (Protein Sciences, Portland, OR). The scaffold was applied to filter and group all of the matching peptides to generate and retain only high confidence IDs while also generating normalized spectral counts (N-SCs) across all samples. The filter cut-off values were set with peptide length (>5 AAs), no peptides with a MH + 1 charge state were included, and peptide probabilities were calculated and set to >80% C.I., with the number of peptides per protein set at 2 or more, and protein probabilities were set to >99% C.I. and an FDR < 1.0. The false discovery rate (FDR) and protein probability^32–34^ were used to statistically validate the proteome datasets. Spectral counting was used for relative quantification across experiments^35,36^, and when relevant, spectral count abundances were then normalized between samples^37^.

### Proteomics Data assessment

The differentially expressed proteins (fold change ± 1.5 fold and p < 0.05) were identified using T-test and used for further analysis. Functional analysis was performed using PANTHER (Protein ANalysis THrough Evolutionary Relationships)^38^ and Ingenuity Pathway Analysis (QIAGEN Inc. MD, USA). The Heat maps were generated using pheatmap package V.1.0.7 in R program.

### Western Blot

Western blot analysis of hippocampal homogenates was done at P14 and young adult age (14 weeks) as described earlier^20^. Briefly, the hippocampus was homogenized in 1 ml of a tissue protein extraction reagent (T-PER, Pierce Biotechnology), centrifuged at 7000 × g for 5 min and the supernatant frozen at −800C until analysis. Protein concentrations were measured using the Bio-Rad Bradford Protein Assay (Bio-Rad, Hercules, CA). Ten µg of protein per lane was fractionated by 10% Tris-Glycine SDS-PAGE electrophoresis, followed by transfer to a PVDF membrane (Millipore, Billerica, MA). Western Blot analysis was done using specific primary antibodies (rabbit polyclonal for total TrkB [abcam, Cambridge, MA], rabbit polyclonal for total AKT [Cell Signaling, Danvers, MA], rabbit monoclonal for total ERK2 [abcam, Cambridge, MA]), and goat polyclonal for β-actin [Santa Cruz Biotechnology, Dallas, Tx]) at 1:1000–2000 dilution for 2 hours at room temperature. The anti-rabbit secondary antibodies [Sigma-Aldrich, St. Louis, MO] for TrkB, AKT, and ERK2 and anti-goat secondary antibody [Sigma-Aldrich, St. Louis, MO] β-actin were used at 1: 10,000 dilution for 1 hour at room temperature. Immunoreactive bands were visualized by treatment with Immun-Star Western blotting detection reagents (Bio-Rad) according to the manufacturer’s instructions. Densitometry was done using a ChemiDoc MP Imaging System (BioRAD, Hercules, CA), normalizing for β-actin in the same lane.

### mRNA isolation

Immediately after harvesting, left hippocampal tissue was flash frozen in an Eppendorf tube with steel beads. Tissue was then homogenized using Qiagen tissue lyser (Qiagen, MD, USA) and RLT + BME buffer solution, and RNA was isolated using Qiagen RNeasy mini RNA isolation kit (Qiagen, MD, USA)^39^.

### mRNA sequencing

Targeted mRNA sequencing was performed on the Illumina NextSeq. 500 as described by the manufacturer (Illumina Inc., San Diego, CA). Briefly, the quality of the total RNA was assessed using the Agilent 2100 Bioanalyzer. RNA with a RNA Integrity Number (RIN) of 7.0 or above was used for sequencing library preparation. Agilent SureSelect Strand-Specific mRNA library kit was used as per the manufacturer’s instructions (Agilent, Santa Clara, CA). Library construction was initiated with two rounds of polyA selection using oligo dT containing magnetic beads. The resulting mRNA was randomly fragmented with cations and heat, which was followed by first strand synthesis using random primers with the inclusion of Actinomycin D (2.4 ng/µL final concentration). Second strand cDNA production was done with standard techniques, and the ends of the resulting cDNA were made blunt. A-tailed and adaptors ligated for indexing to allow for multiplexing during sequencing. The cDNA libraries were quantitated using qPCR in a Roche LightCycler 480 with the Kapa Biosystems kit for Illumina library quantitation (Kapa Biosystems, Woburn, MA) before cluster generation. Cluster generation was performed according to the manufacturer’s recommendations for onboard clustering (Illumina). Paired-end 75 bp sequencing runs was completed to allow for better alignment of the sequences to the reference genome.

### Analysis of Oxidative Stress

The whole hippocampus was homogenized and analyzed for malondialdehyde (MDA) adducts by ELISA as per manufacturer’s protocol (Cell Biolabs, San Diego, CA). DNA was isolated from the whole hippocampus using a Qiagen DNeasy Blood and Tissue kit (Qiagen, Valencia, CA) and analyzed for 8-OH deoxyguanosine (8-OHdG) by ELISA (Cell Biolabs).

### Histology

Mice were sacrificed at P14, and the brains were stored in formalin overnight. Brains were then transferred to 30% sucrose followed by antifreeze solution (ethylene glycol + 30% sucrose in 0.1 M phosphate buffer) and stored at −20 °C. As described previously^19^, 6 series of 30 µm thick brain sections were stained using primary antibodies for proliferating cell nuclear antigen for proliferating cells (Santa Cruz Biotechnology, Dallas,Tx), Glial fibrillary acidic protein (GFAP) for reactive astrocytes (Sigma-Aldrich, St. Louis, MO), and 2′,3′-Cyclic-nucleotide 3′-phosphodiesterase (CNPase) for myelin protein (abcam, Cambridge, MA). Nonspecific IgG and omission of primary antibody were used as controls for staining specificity.

Photomicrographs of CA1 and dentate gyrus regions of the hippocampi were taken using 10x objective. Using MetaMorph v.6.2 software, the number of cells stained for PCNA were counted (non-stereological method), while the area stained for GFAP and CNPase were analyzed using intensity thresholding and expressed as a percentage of total area as described previously^19^.

## Results

### Targeted Proteomics and Transcriptomics

Transcriptomic data are available on GEO (https://www.ncbi.nlm.nih.gov/geo/) with accession number GSE111189.

#### Effect of Hyperoxia Exposure on Hippocampal mGlu7, NMDA, AMPA, and GABA Receptors, Synaptic Proteins, and Synaptic SNARE Molecules

P14 mice exposed to neonatal hyperoxia had reduced amounts of hippocampal glutamate receptor metabotropic 7 (mGLU7) protein but with no change at the mRNA level compared to air-exposed mice (Table 1). The protein levels of NMDA receptor subunit 1 (GRIN1), NMDA receptor subunit 2B (GRIN2B), AMPA receptor subunit 1 (GLUR1), and AMPA receptor subunit 2 (GluR2) were comparable between air- and hyperoxia-exposed mice (Table 1). However, hyperoxia-exposed mice had increased GRIN1, GLUR1, and decreased GRIN2B mRNA levels compared to the air-exposed mice (Table 1). Among gamma-aminobutyric acid type A receptor (GABA_A_) receptor subunits, the protein levels of α3 (GABRA3), α5 (GABRA5), and ɤ2 (GABRG2) subunits were increased in hyperoxia-exposed mice. While the mRNA of α5 GABA_A_ receptor subunit was not significantly different in hyperoxia-exposed mice, the levels of α3 and ɤ2 were decreased in hyperoxia-exposed mice (Table 1). Protein levels of GABA_A_ α1 (GABRA1) and β3 (GABRB3) subunits were not statistically different. However, compared to the air-exposed mice, GABA_A_ α1 mRNA expression was reduced in hyperoxia-exposed mice. The protein and gene expression levels of gamma-aminobutyric acid type B receptor (GABA_B_) subunit 2 were comparable between the groups (Table 1).

**Table 1:** Effect of Hyperoxia Exposure on Hippocampal mGlu7, NMDA, AMPA and GABA receptors, Synaptic Proteins and Synaptic Snare molecules (n = 5 in Air group, 6 in Hyperoxia group) and Corresponding Gene Expression (n = 3 in Air group, 3 in Hyperoxia group).

Molecule (Symbol)	Protein Log Fold Change in Hyperoxia (vs. Air)	P value	Gene Expression Log Fold Change in Hyperoxia (vs. Air)	P value
**Presynaptic Glutamate receptor**				
Glutamate receptor, metabotropic 7 (mGlu7)	−1.44	**0.01**	−0.02	0.8
**N-Methyl-D-aspartic acid (NMDA) receptors**				
NMDA receptor subunit 1 (GRIN1)	+0.35	0.62	+0.55	**2.02E-07**
NMDA receptor subunit 2B (GRIN2B)	−0.07	0.83	−0.31	**0.05**
**α-amino-3-hydroxy-5-methyl-4-isoxazolepropionic acid (AMPA) receptors**				
AMPA receptor subunit 1 (GlUR1)	−0.22	0.79	+0.53	**0.0001**
AMPA receptor subunit 2 (GlUR2)	−0.01	0.97	−0.19	0.17
**Gamma-aminobutyric acid (GABA) receptors**				
GABA type A receptor α1 Subunit (GABRA1)	+0.54	0.36	−0.24	**0.04**
GABA type A receptor α3 subunit (GABRA3)	+1.80	**0.007**	−0.85	**2.26E-08**
GABA type A receptor α5 subunit (GABRA5)	+0.68	**0.04**	+0.14	0.17
GABA type A receptor β3 Subunit (GABRB3)	+0.02	0.94	−0.13	0.13
GABA type A receptor ɤ2 subunit (GABRG2)	+0.92	**0.05**	−0.20	**0.04**
GABA type B receptor Subunit 2 (GABBR2)	+0.06	0.92	−0.02	0.74
**Synaptic Proteins**				
Gephyrin (GPHN)	+0.55	0.40	+0.14	0.26
Synaptopodin (SYNPO)	+0.01	0.98	**+0.48**	**6E-06**
Protein interacting with PRKCA 1 (PICK1)	+0.24	0.65	+0.18	0.17
Vesicular glutamate transporter 1 (VGLUT1)	−0.30	0.56	**+0.33**	**5.3E-05**
Leucine rich glioma inactivated 1 (LGI1)	−0.75	0.27	+0.17	0.19
Agrin (AGRN)	+0.82	0.22	−0.14	0.33
Glutamate receptor interacting protein 1 (GRIP1)	+0.07	0.93	+0.14	0.48
Cadherin 2 (CDH2)	+0.97	0.11	+0.19	0.23
Post-synaptic density protein 95 (PSD95)	+0.29	0.70	**+0.31**	**0.005**
**Synaptic SNARE Molecules**				
Syntaxin 7 (STX7)	+0.62	0.35	−0.15	0.18
Syntaxin 1A (STX1A)	+0.06	0.93	−0.34	**0.05**
Syntaxin 12 (STX12)	+0.75	0.35	−0.19	**0.05**
Syntaxin 6 (STX6)	+1.52	**0.03**	**+0.26**	**0.001**
Syntaxin 8 (STX8)	+1.08	0.09	+0.07	0.62
Synaptosome associated protein 29 (SNAP29)	+0.11	0.86	−0.18	0.13
Synaptosome associated protein 23 (SNAP23)	+0.57	0.42	−0.16	0.32

While the mRNA expression levels of synaptopodin (SYNPO), vesicular glutamate transporter 1 (VGLUT1), and post-synaptic density protein 95 (PSD95) were increased in hyperoxia-exposed mice, corresponding protein levels were not statistically different (Table 1). The protein and mRNA expression levels of gephyrin (GPHN), protein interacting with PRKCA 1 (PICK1), leucine rich glioma inactivated 1 (LGI1), agrin (AGRN), glutamate receptor interacting protein 1 (GRIP1), and cadherin 2 (CDH2) were also not different between the groups (Table 1).

Analysis of syntaxins indicated that the protein and mRNA expression levels of syntaxin 7 (STX7), and syntaxin 8 (STX8) were similar between air- and hyperoxia-exposed mice (Table 1). The syntaxin 6 (STX6) protein and mRNA levels were increased in hyperoxia-exposed mice. No difference in the protein levels of syntaxin 1A (STX1A) and syntaxin 12 (STX12) were observed between the groups (Table 1). However, the mRNA levels of STX1A and STX12 were decreased in hyperoxia-exposed mice compared to air-exposed mice. The protein and mRNA levels of synaptosome associated protein 29 (SNAP29), and synaptosome associated protein 23 (SNAP23) were similar between the groups (Table 1).

#### Effect of Hyperoxia Exposure on Hippocampal Calmodulin, TrkB/ERK/AKT Signaling Pathways, and Transcription Factors

Protein levels of calcium/calmodulin-dependent protein kinase (CAMK) type I and II and their subunits were comparable between the groups (Table 2). However, increased CAMKI, CAMKIIA, and CAMKIIB gene expression levels and decreased CAMKIID and CAMKIIG were noted in hyperoxia-exposed compared to air-exposed mice.

**Table 2:** Effect of Hyperoxia on Calmodulin, TrkB/ERK/PI3K Signaling Pathways, and Transcription Factors (n = 5 in Air group, 6 in Hyperoxia group) and Corresponding Gene Expressions (n = 3 in Air group, 3 in Hyperoxia group).

Molecule (Symbol)	Protein Log Fold Change in Hyperoxia (vs. Air)	P value	Gene Expression Log Fold Change in Hyperoxia (vs. Air)	P value
**Calmodulins**				
Calcium/calmodulin-dependent protein kinase type I (CAMKI)	−0.764	0.06	**+0.36**	**0.01**
Calcium/calmodulin-dependent protein kinase type II α (CAMKIIA)	−0.62	0.20	**+0.46**	**8.14E-05**
Calcium/calmodulin-dependent protein kinase type II β (CAMKIIB)	−0.31	0.53	**+0.52**	**1.05E-05**
Calcium/calmodulin-dependent protein kinase type II δ (CAMKIID)	+0.13	0.83	**−0.83**	**7.17E-05**
Calcium/calmodulin-dependent protein kinase type II ɤ (CAMKIIG)	−0.070	0.86	**−0.36**	**0.0001**
**TrKB/ERK/PI3K Pathway**				
Tropomyosin receptor kinase B (TrkB)	−1.09	**0.04**	−0.04	0.76
Protein kinase C α (PKCA)	+0.01	0.98	+0.25	0.06
Protein kinase C β (PKCB)	+1.12	0.17	−0.27	**0.009**
Protein kinase C ɤ (PKCG)	−0.22	0.45	**+0.56**	**8.46E-07**
Mitogen-activated protein kinase Kinase 1(MEK1)	−0.26	0.57	+0.13	0.11
Mitogen-activated protein kinase Kinase 2(MEK2)	+0.53	0.35	−0.07	0.5
Extracellular signal-regulated kinase 1 (ERK1)	+0.58	0.36	+0.18	0.12
Extracellular Signal-Regulated Kinase 2 (ERK2)	**−1.33**	**0.02**	**+0.15**	**0.03**
Phosphoinositide 3-kinase (PI3K)	+0.25	0.13	**−0.3**	**0.0007**
Phosphatase and tensin homolog (PTEN)	+0.71	**0.03**	**−0.63**	**3.66E-08**
Protein kinase B (PKB-AKT)	**−1.02**	**0.02**	**−0.70**	**1.81E-09**
AKT interacting protein (AKTIP)	**−1.27**	**0.03**	**−0.17**	**0.04**
Mechanistic target of rapamycin (mTOR)	+0.50	0.48	**−0.33**	**3.55E-05**
Mechanistic target of rapamycin complex 1 (mTORC1)	**−0.98**	**0.01**	**+0.18**	**0.05**
Brain-derived neurotrophic factor (BDNF)	Not Detected	—	**+0.40**	**0.0004**
Ribosomal protein S6 RPS6	**−0.89**	**0.04**	**+0.20**	**0.04**
CAMP Responsive Element Binding Protein 1 (CREB1)	Not Detected	—	+0.006	0.97
Eukaryotic translation initiation factor 4e (EIF4E)	**−1.18**	**0.008**	**+0.15**	**0.05**
**Transcription Factors Involved in Long-Term Memory and Synaptic Plasticity**				
Jun proto-oncogene (JUN)	Not Detected	NA	**+0.67**	**1.82E-06**
Nuclear factor kappa B (NFKB)	Not Detected	NA	−0.09	0.52
Activating transcription factor 4 (ATF4)	Not Detected	NA	**+0.29**	**0.02**
**Transcription Factors Involved in Development**				
Paired Box 6 (PAX6)	Not Detected	NA	+0.18	0.26
T-box, brain 1 (TBR1)	Not Detected	NA	−0.005	0.98
T-box, brain 2 (TBR2)	Not Detected	NA	**+0.59**	**0.01**
SRY-box 2 (SOX2)	Not Detected	NA	−0.18	0.30
Prospero homeobox 1 (PROX1)	Not Detected	NA	+0.26	0.35
Forkhead box G1 (FOXG1)	Not Detected	NA	+0.24	0.11
**Immediate-Early Genes**				
Fos proto-oncogene (FOS)	Not Detected	NA	+0.28	0.11
Activity-Regulated Cytoskeleton-Associated Protein (ARC)	Not Detected	NA	**+1.51**	**5E-32**
Early Growth Response 1 (EGR1)	Not Detected	NA	**+0.78**	**2.2E-06**
CCAAT/enhancer binding protein (C/EBP)	Not Detected	NA	**+0.96**	**4.72E-07**

Hyperoxia-exposed mice had reduced hippocampal TrkB protein with no change in corresponding mRNA levels. The protein levels of protein kinase C subunits (α, β, ɤ), downstream proteins of TrKB signaling pathway, were similar between air-exposed and hyperoxia-exposed mice (Table 2). The gene expression levels of protein kinase C subunits α was comparable between the groups but the gene expression level of protein kinase C β subunit was decreased and protein kinase C ɤ was increased in the hyperoxia group. The protein and gene expression levels of mitogen-activated protein kinase 1, 2 (MEK1, MEK2), and ERK1 were not different between the groups (Table 2). However, the protein level of ERK2 was decreased, and its corresponding gene expression level was increased in hyperoxia-exposed mice (Table 2). While the mRNA of PI3K was decreased in hyperoxia-exposed mice, the protein level was comparable to that of air-exposed mice.

Hyperoxia-exposed mice had lower protein amounts of protein kinase B (AKT), AKT interacting protein, the mechanistic target of rapamycin complex 1 (mTORC1), ribosomal protein S6, and eukaryotic translation initiation factor 4e (EIF4E) compared to air-exposed mice (Table 2). Similar to that of protein levels, the mRNA of protein kinase B (AKT) and AKT interacting protein were reduced in the hyperoxia group. However, the mRNA of mechanistic target of rapamycin complex 1 (mTORC1), ribosomal protein S6, and eukaryotic translation initiation factor 4e (EIF4E) were increased with hyperoxia. In addition, phosphatase and tensin homolog (PTEN) expression was increased in hyperoxia-exposed mice. The protein level of mechanistic target of rapamycin (mTOR) was similar between air-and hyperoxia-exposed groups (Table 2). However, the mRNA level of mTOR was decreased in hyperoxia-exposed mice compared to air-exposed mice. While the mRNA level of brain-derived neurotrophic factor (BDNF) was increased in hyperoxia-exposed mice, BDNF protein was not detected in either group. Our analysis did not detect cAMP response elements (CREB) or phosphorylated CREB in both groups.

Transcription factor proteins involved in long-term memory and synaptic plasticity (jun proto-oncogene [JUN], nuclear factor kappa B [NFKB], and activating transcription factor 4 [ATF4]) and neurodevelopment (paired Box 6 [PAX6], T-box, brain 1 [TBR1], T-box, brain 2 [TBR2], SRY-box 2 [SOX2], prospero homeobox 1 [PROX1], and forkhead box G1 [FOXG1]) were not detected in either air- and hyperoxia-exposed mice, probably due to their very low relative concentrations (Table 2). However, mRNA levels of JUN, ATF4, and TBR2 were increased in hyperoxia-exposed mice while mRNA levels of NFKB, PAX6, TBR1, SOX2, PROX1, and FOXG1 were not different between the groups (Table 2). Among the immediate early genes, mRNA of activity-regulated cytoskeleton-associated protein (ARC), early growth response 1 (EGR1), and CCAAT/enhancer binding protein (C/EBP) were decreased with hyperoxia. No difference in the mRNA expression level of fos proto-oncogene (FOS) was observed between the groups (Table 2).

### Bioinformatic Analysis of Differentially Expressed Hippocampal Proteins

#### Differentially Expressed Hippocampal Proteins Following Hyperoxia

**Figure 1:**
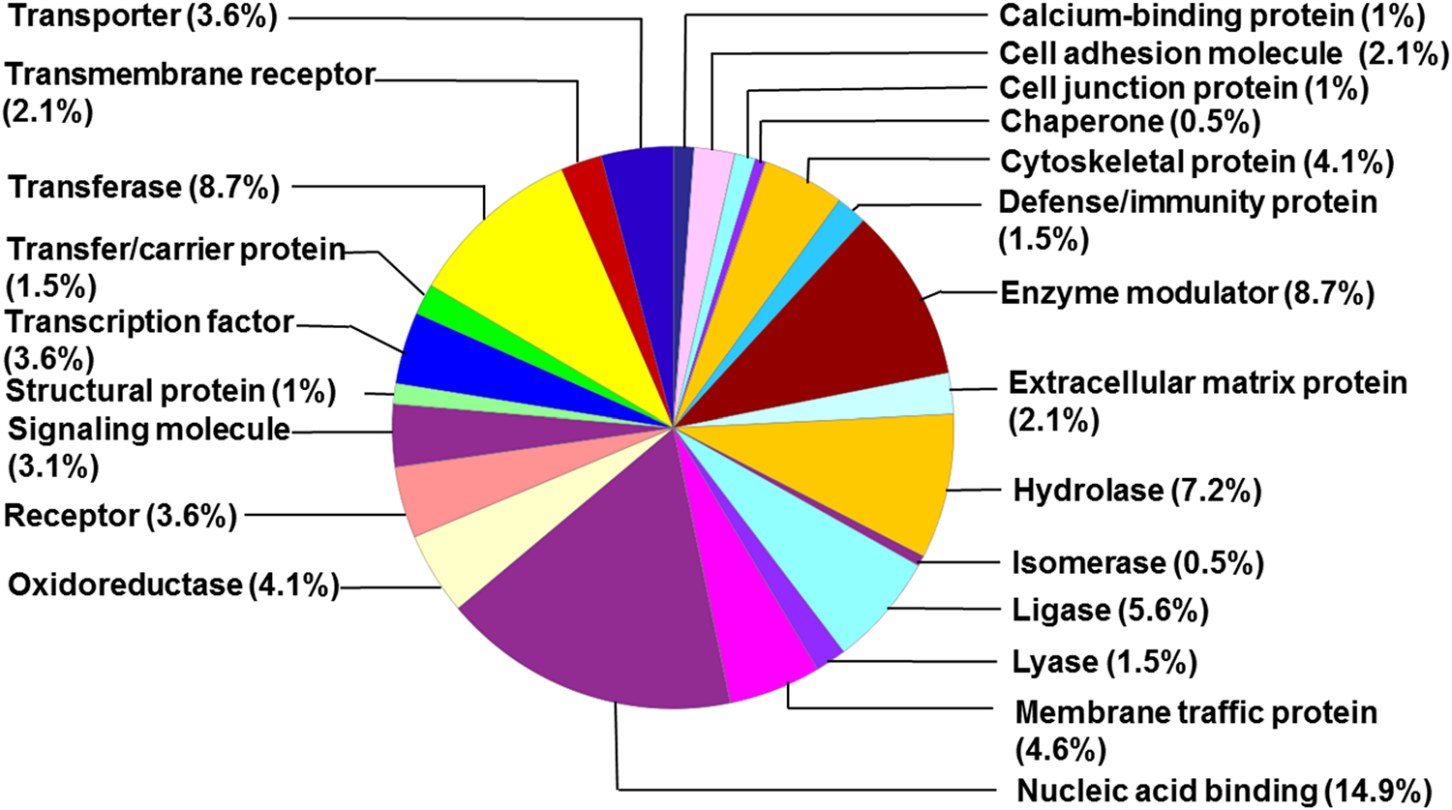
Distribution of upregulated hippocampal proteins by class in the hyperoxia-exposed group.

**Figure 2:**
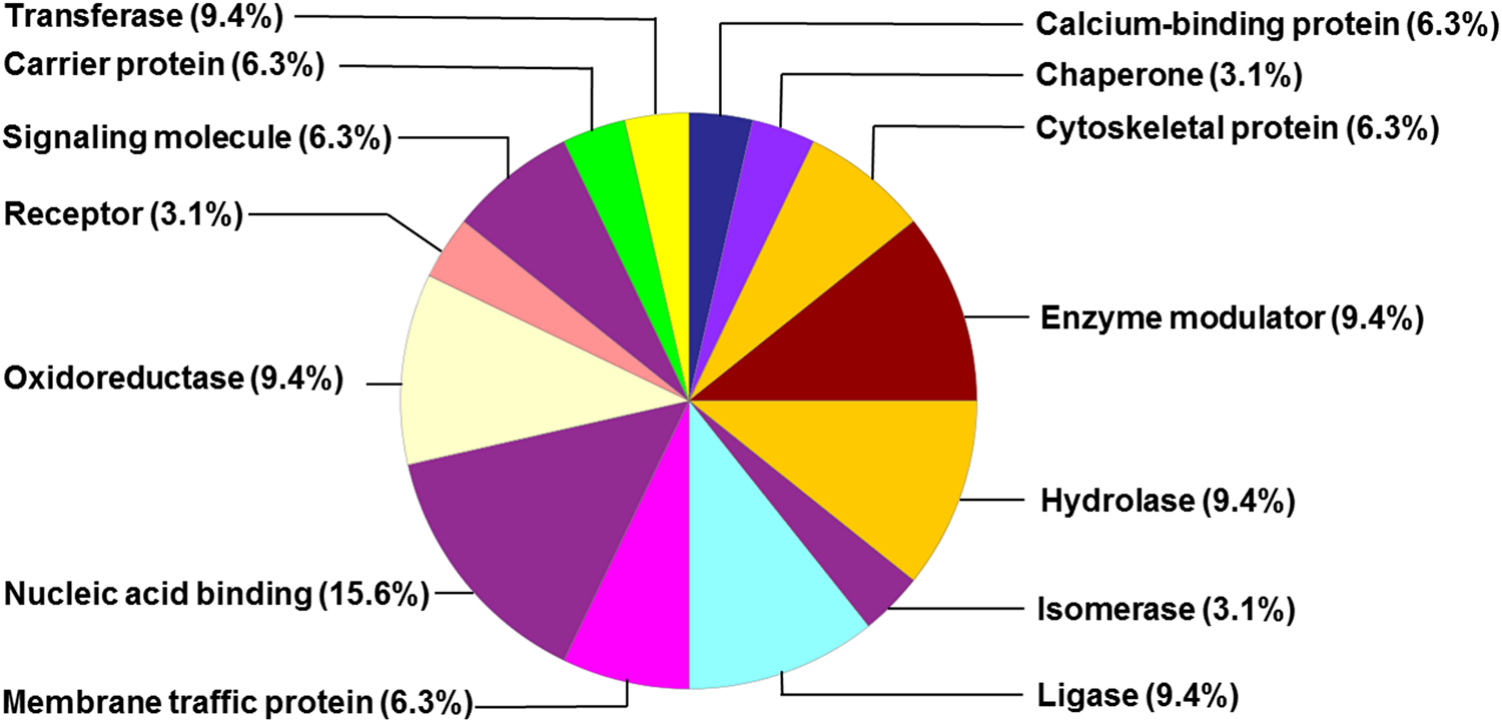
Distribution of downregulated hippocampal proteins by class in the hyperoxia-exposed group.

Using a cut-off of ±1.5 fold-change with P-value < 0.05 (by analysis of variance), and a false discovery rate of 5%, we identified 225 hippocampal proteins that were differentially expressed in the hyperoxia-exposed mice compared to air-exposed mice. Of these 225 proteins, 193 proteins were increased, and 32 were decreased following hyperoxia. The heat map of the differentially expressed proteins is shown in Supplemental Fig. 1. The top 10 differentially expressed proteins in the oxygen-exposed group are listed in Table 3. The full list of upregulated proteins in hyperoxia-exposed mice and corresponding gene expression is shown in Supplemental Table 1. The list of downregulated proteins in hyperoxia-exposed mice and corresponding gene expression is shown in Supplemental Table 2. The list of differentially expressed genes in hyperoxia-exposed mice is shown in Supplemental Tables 3 and 4. The protein classes that are upregulated and downregulated by oxygen exposure are shown in Figs 1 and 2, respectively. The upstream and downstream signaling networks of AKT and ERK1/2 identified as altered by proteomic analysis are shown in Supplemental Figs 2 and 3, respectively.

**Table 3:** Top 10 Differentially Expressed Proteins in Hyperoxia-Exposed Mice (n = 5–6 per group, P vs. Air group).

Protein (Symbol)	Log Fold Change in Hyperoxia Group	P value
**Up-regulated Proteins**		
Proline rich coiled-coil 2 C (PRRC2C)	+2.42	0.0008
Nucleobindin 1 (NUCB1)	+2.32	0.006
Zinc finger protein 638 (ZNF638)	+2.32	0.003
Microtubule Affinity Regulating Kinase 2 (MARK2)	+2.20	0.0001
SWI/SNF-related matrix-associated actin-dependent regulator of chromatin subfamily D member 3 (SMARCD3)	+2.19	0.006
**Down-regulated Proteins**		
Teneurin transmembrane protein 2 (TENM2)	−1.86	0.01
Ankyrin 2 (ANK2)	−1.75	0.03
Late endosomal/lysosomal adaptor, MAPK and MTOR activator 3 (LAMTOR3)	−1.69	0.02
Embryonic lethal, abnormal vision like 1 (ELAVL1)	−1.68	0.02
Ubiquinol-cytochrome c reductase, complex III subunit 10 (UQCR10)	−1.67	0.01

#### Hippocampal Biological Processes Modulated by Hyperoxia Exposure

**Figure 3:**
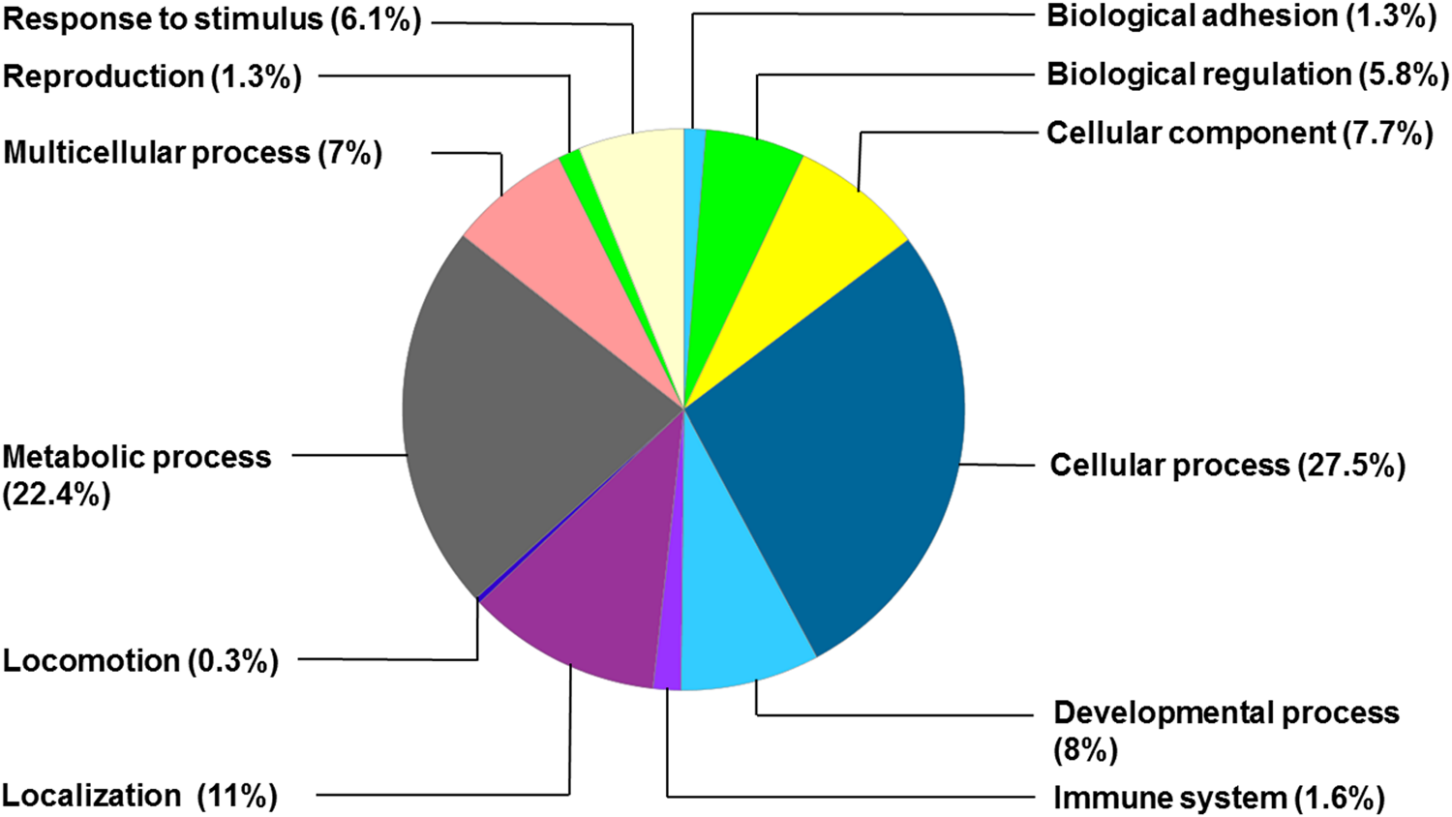
Distribution of upregulated hippocampal proteins by biological processes in the hyperoxia-exposed group.

**Figure 4:**
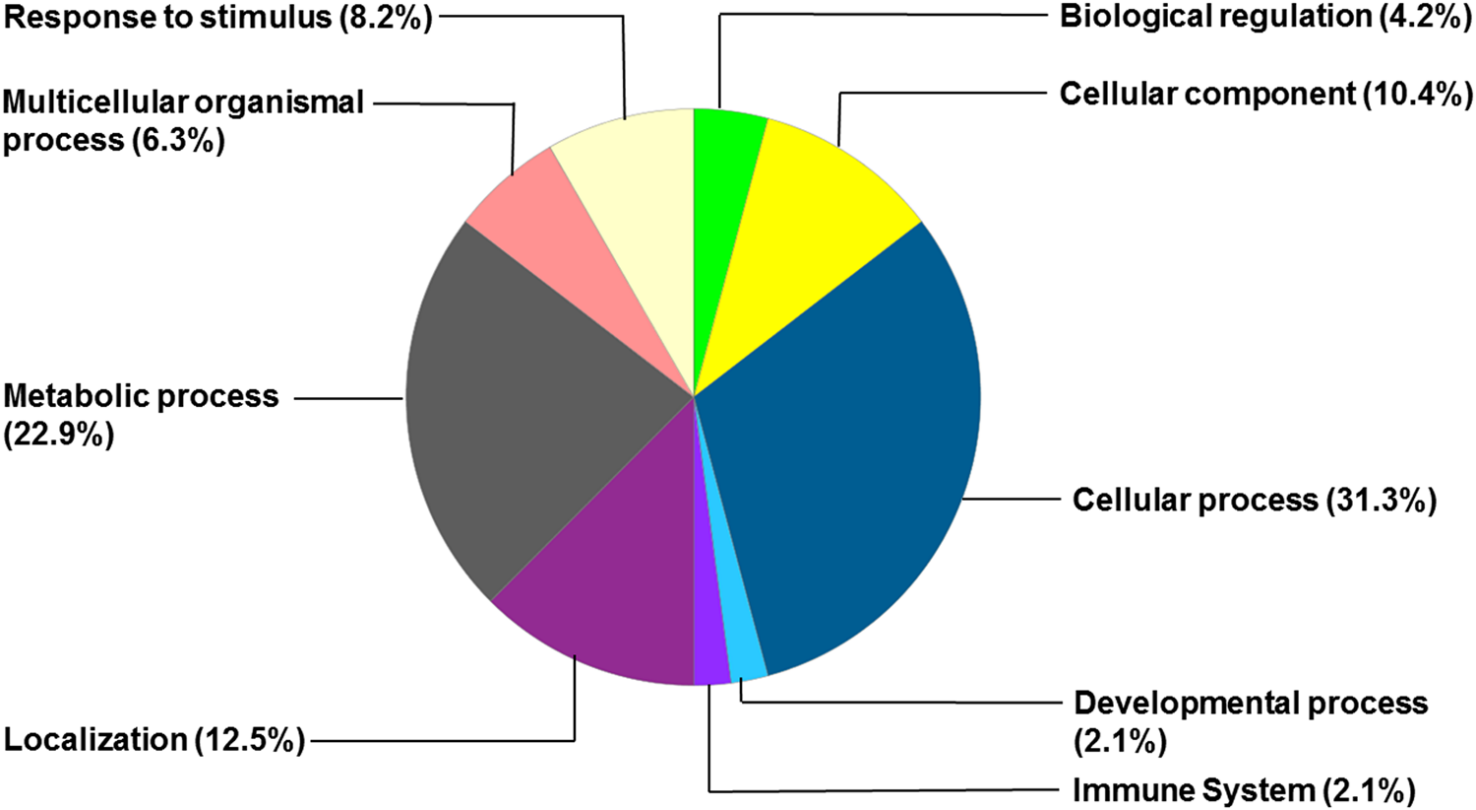
Distribution of downregulated hippocampal proteins by biological processes in the hyperoxia-exposed group.

Differentially expressed hippocampal proteins were predominantly involved in the cellular process, metabolic process, and protein localization (Figs 3 and 4). Among the upregulated proteins, functions of 54 (27.5%) proteins were related to cellular process, 44 (22.4%) were proteins related to metabolic process, and 21 (11%) were proteins related to localization process (Fig. 3). Among the downregulated proteins, functions of 10 (31.3%) proteins were related to cellular process, 7 (22.9%) were proteins related to metabolic process, and 4 (12.5%) were proteins related to localization process (Fig. 4).

#### Top Canonical Hippocampal Pathways Regulated by Hyperoxia Exposure

The top canonical pathways that were impacted by oxygen exposure are listed in Table 4. Bioinformatic analysis of differentially expressed proteins predicated that mitochondria function, eukaryotic initiation factor 2 (EIF2) signaling, oxidative phosphorylation, regulation of eukaryotic initiation factor 4 (EIF4)/ribosomal protein S6 kinase beta-1 (S6K1), peroxisome proliferator-activated receptor (PPAR)/retinoid X receptor (RXR) activation, nuclear factor like 2 (NRF2)-mediated oxidative stress response, and protein ubiquitination pathways were impacted in the hyperoxia-exposed group. Eighty-nine proteins were related to mitochondrial function, 102 related to EIF2 signaling, 59 related to oxidative phosphorylation, 73 proteins related to regulations of eIF4 and S6K1 signaling, 52 PPAR/RXR activation, 66 NRF2- mediated oxidative stress response, and 94 proteins related to protein ubiquitination pathway were differentially expressed in the hyperoxia-exposed group.

**Table 4:** Top Canonical Pathways Involved in Hyperoxia-Exposed Group by Ingenuity Pathway Analysis (n = 5 in Air group, 6 in Hyperoxia group, P = Hyperoxia vs. Air group).

Name	P-Value	Overlap (percentage; number of proteins differentially expressed/number of proteins in pathway)
Mitochondrial dysfunction	2.94E-33	52.0%, 89/171
Regulation of eukaryotic initiation factor 2 (EIF2) signaling	3.66E-32	46.2%, 102/221
Oxidative phosphorylation	1.16E-23	54.1%, 59/109
Regulation eukaryotic initiation factor 4 (EIF4) and ribosomal protein S6 kinase beta-1 (S6K1) signaling	1.23E-23	46.5%, 73/157
PPAR/RXR activation	9.53E-08	28.4%, 52/183
NRF2-mediated oxidative stress response	1.96E-08	26.9%, 66/245
Protein ubiquitination pathway	1.02E-19	35.5%, 94/265

#### Top Hippocampal Upstream Regulators Involved in Hyperoxia-Exposed Mice

The top upstream regulators for the differentially expressed proteins in the hyperoxia-exposed mice and corresponding gene expressions are shown in Table 5. Bioinformatic analysis of differentially expressed hippocampal proteins predicted microtubule-associated protein tau (MAPT), rapamycin-insensitive companion of mammalian target of rapamycin (RICTOR), amyloid precursor protein (APP), presenilin 1 (PSEN1) and MYC gene as the top upstream regulators associated with hyperoxia exposure (Table 5). Based on the differentially expressed genes, the bioinformatic analysis predicted CAMP Responsive Element Binding Protein 1 (CREB1), forskolin (cyclic AMP upregulator), calcium ion, bradykinin receptor B1 (BDKRB1), and prodynorphin (PDYN) as the top upstream regulators associated with hyperoxia exposure (Table 5).

**Table 5:** The Top Upstream Regulators in the Hyperoxia-Exposed Mice by Ingenuity Pathway Analysis (n = 5–6 per group, P vs. Air group).

Symbol	Molecule	P-value of overlap	Protein Log Fold Change in Hyperoxia (vs. Air)	P value	Gene Expression Log Fold Change in Hyperoxia (vs. Air)	P value
**Top Upstream Regulators Using Proteomics**						
MAPT	Microtubule-associated protein tau	4.60E-64	0.22	0.71	−0.03	0.82
RICTOR	Rapamycin-insensitive companion of mTOR	4.33E-59	NA	NA	−0.40	0.02
APP	Amyloid precursor protein	5.35E-40	0.02	0.96	−0.05	0.69
PSEN1	Presenilin-1	2.03E-39	NA	NA	+0.01	0.92
MYC	Myc proto-oncogene protein	3.44E-36	−0.48	0.29	+0.20	0.13
**Top Upstream Regulators Using Transcriptomics**						
CREB1	CAMP Responsive Element Binding Protein 1	1.32E-06	NA	NA	+0.006	0.970
Forskolin	(cyclic AMP upregulator)	3.60E-05	NA	NA	—	—
Ca2+	Calcium Ion	5.43E-05	NA	NA	—	—
BDKRB1	Bradykinin Receptor B1	2.09E-04	NA	NA	−0.41	0.13
PDYN	Prodynorphin	4.98E-04	NA	NA	+0.08	0.64

#### Effect of Hyperoxia on Hippocampal Levels of TrkB, AKT, and ERK2 at P14 and in Young Adult Mice (14 weeks of age)

**Figure 5:**
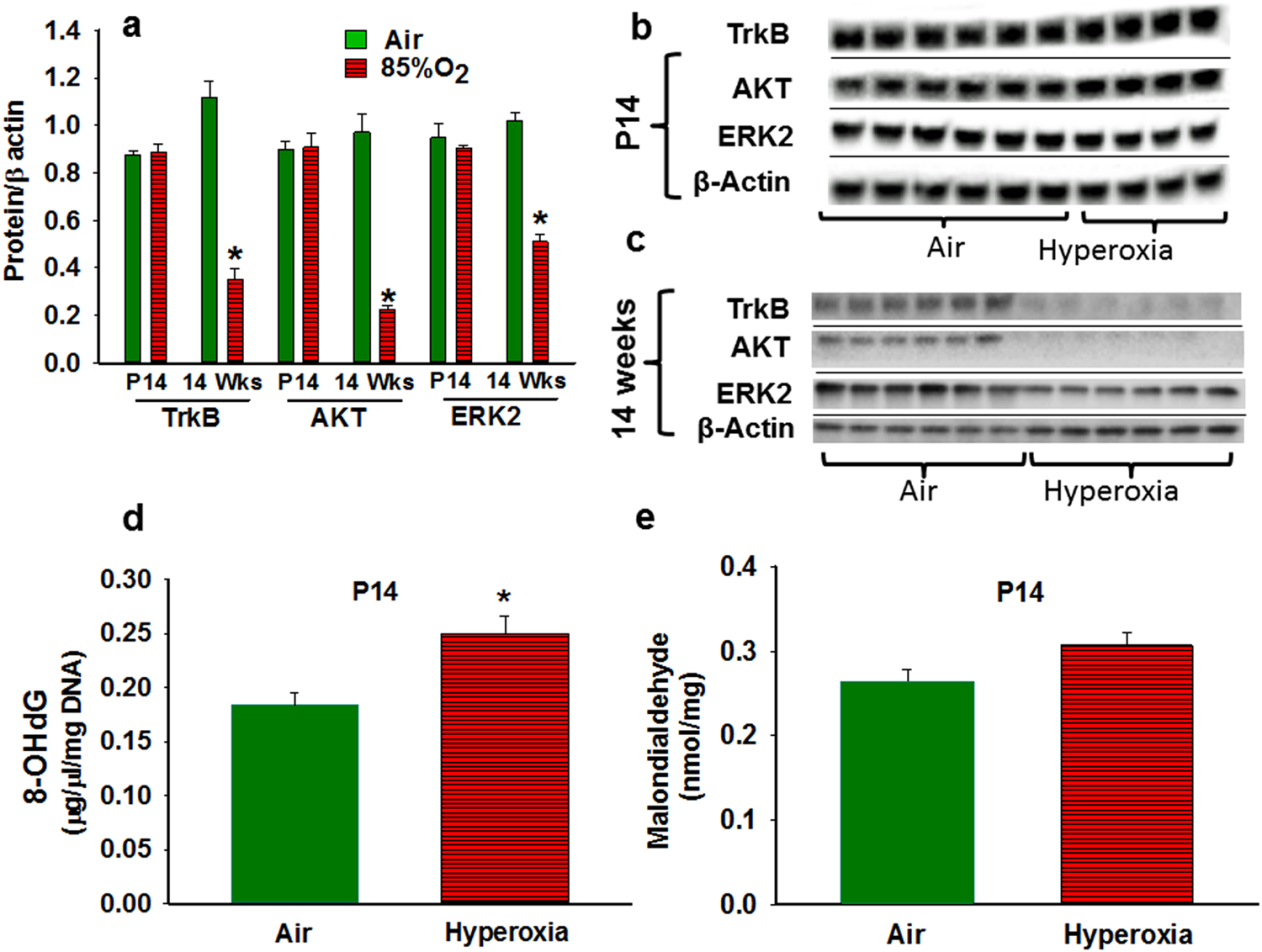
Effect hyperoxia on whole hippocampal TrkB, AKT, and ERK2 concentrations and oxidative stress markers. (**a**) Graphical representative of Western blot results from hippocampal samples of P14 and 14 weeks old mice. At P14, total TrkB, AKT, and ERK2 levels were not significantly different between air-and hyperoxia-exposed mice. At 14 weeks, TrkB, AKT, and ERK2 levels were decreased in the hyperoxia group. (**b**) Representative of hippocampal western blots from P14 mice exposed to Air (n = 6) or Hyperoxia (n = 4). The blots were obtained from different gels and pictures were taken with the same exposure. (**c**) Representative of hippocampal western blots from 14-week-old mice exposed to Air (n=) or Hyperoxia (n = 6). The blots were obtained from different gels and pictures were taken with the same exposure. (**d**) Graphical representative of 8-OHdG (oxidative DNA damage marker) ELISA results from P14 mice exposed to Air (n=) or Hyperoxia (n = 6). Hyperoxia-exposed mice had increased 8-OHdG in the hippocampus. (**e**) Graphical representative of malodialdehyde (MDA adducts: lipid peroxidation marker) ELISA results from P14 mice exposed to Air (n = ) or Hyperoxia (n = 6). No difference in MDA adducts were observed between the groups. In the panels a, d, and e; Air: solid green bars, Hyperoxia: solid red bars with horizontal stripes. *Represents p < 0.05; Air vs. Hyperoxia.

Protein levels of total TrkB, total AKT and total ERK2 by immunoblotting were comparable at P14 in both hyperoxia-and air exposed group (Fig. 5a,b). However, at 14 weeks, the protein levels of total TrkB, total AKT, and total ERK2 were reduced in hyperoxia-exposed mice compared to air-exposed mice (Fig. 5a,c).

#### Effect of Hyperoxia on Oxidative Stress Markers, Proliferating cells, Reactive Astrocytes and Myelin Protein

**Figure 6:**
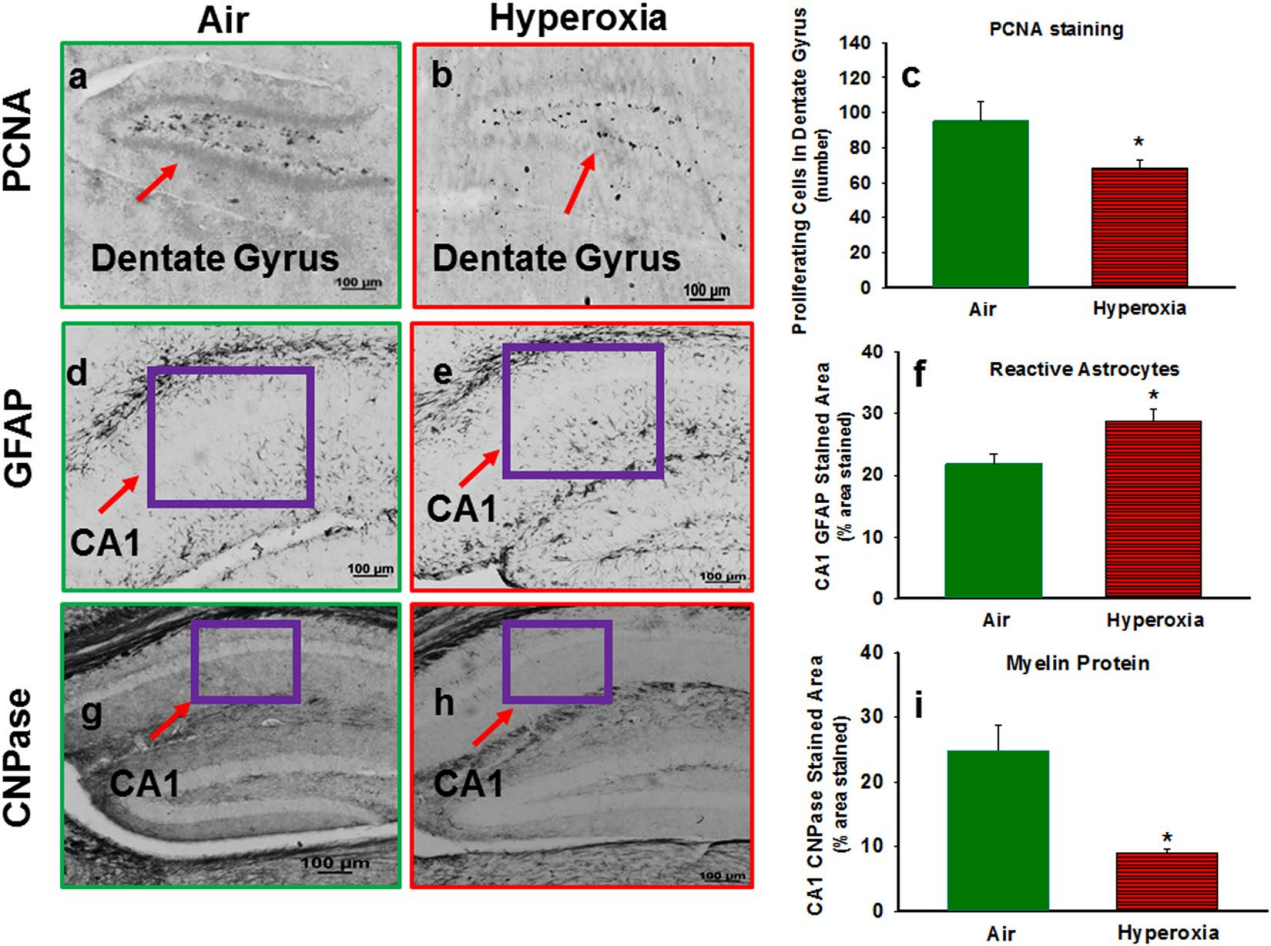
Histological assessment of effect hyperoxia on cell proliferation, reactive astrocytes and myelin protein. Photomicrographs of PCNA stained brain sections from P14 mice exposed to Air (**a**) or Hyperoxia (**b**). (**c**) Graphical representation of PCNA staining results; hyperoxia-exposed mice had a reduction in the number of proliferative cells in the dentate gyrus. Photomicrographs of GFAP stained brain sections from P14 mice exposed to Air (**d**) or Hyperoxia (**e**). (**f**) Graphical of representation GFAP staining results; hyperoxia-exposed mice had an increased in the percentage of GFAP stained area in the CA1 region. Photomicrographs of CNPase stained brain sections from P14 mice exposed to Air (**g**) or Hyperoxia (**h**). (**i**) Graphical representation of CNAPase staining results; hyperoxia exposed mice had decreased in the percentage of CNPase stained area in the CA1 region of the hippocampus. In the panels (c,f), and (i); Air: solid green bars, Hyperoxia: solid red bars with horizontal stripes. *Represents p < 0.05; Air vs. Hyperoxia.

Hyperoxia-exposed mice had increased 8-OHdG in the hippocampus (Fig. 5d). No difference in MDA adducts were observed between the groups (Fig. 5e). Compared to room air-exposed mice, hyperoxia-exposed mice had a reduction in the number of proliferative cells in the dentate gyrus (Fig. 6c). Hyperoxia-exposed mice had an increased in the percentage of GFAP stained area in the CA1 region (Fig. 6f) and decreased in the percentage of CNPase stained area in the CA1 region of the hippocampus (Fig. 6i) compared to the room-air exposed mice.

## Discussion

This is the first animal study to demonstrate the impact of hyperoxia exposure on developing hippocampal signaling pathways and proteins required for learning and memory. Our novel observations were that supraphysiological oxygen exposure during a critical developmental period alters the levels of ligand-gated ion channels and signaling molecules that are essential for hippocampal-dependent learning and memory. Our proteomic analysis predicts that early life oxygen exposure results in hippocampal mitochondrial dysfunction and impairment of global protein synthesis and translation. These observations are clinically important because survivors following extreme preterm birth are at high risk of reduced hippocampal volume associated with memory and learning deficits, which is simulated by our animal model of neonatal hyperoxia exposure.

Our study has the strength of unbiased proteomic analysis of hippocampal tissue, using highly sensitive mass spectrometric methods, enabling simultaneous evaluation of multiple pathways and signaling molecules, rather than being limited to a few pre-selected targets and less quantitative methods such as immunoblotting. In addition to protein determination, we also measured the gene expression of molecules that are essential for learning and memory. The use of a novel newborn mouse model with a clear phenotype following oxidative stress injury is also a strength. This newborn mouse model can provide a framework for experimental investigation by which cellular and molecular therapeutic targets for oxidative stress-induced developmental brain disorders can be determined. This mouse model also mimics the poor initial growth and other organ system dysfunction seen in survivors of extreme preterm birth, including cardiopulmonary abnormalities. Hyperoxia-exposed mice had poor weight gain soon after hyperoxia exposure, but these differences disappeared when mice were evaluated at 8 weeks^19^. Hyperoxia-exposed mice also had abnormalities in lung structure and function which persisted into adult life^40^ despite no difference in pulse oximetry in ambient air observed at P14.

There are also certain limitations to this study. The protein analysis was performed from samples of whole right hippocampus instead from specific hippocampal subfields (such CA1, CA2, CA3, and dentate gyrus) which are known to play variable roles in learning and memory. The small size of the hippocampus at P14 was a major limitation and prevented analysis of specific hippocampal subfields. Even though the mass spectroscopic analysis we used is highly sensitive, the levels of brain derived neurotrophic factor (BDNF), and phospholipase C were undetectable in both groups. Furthermore, it is impossible to determine whether these oxygen-induced proteins changes were predominantly derived from neurons or glial cells or combination of both. It is highly likely that other regions of the brain (e.g. cerebellum, amygdala, corpus callosum and white matter tracts) are also impacted by hyperoxia exposure^41–43^. Our focus has so far been on the hippocampus due to its relevance to spatial and recognition memory, which we found were highly affected in our model. It is possible that connectivity of the hippocampus with other brain regions (e.g. entorhinal cortex) is also affected.

Mouse models are commonly used animal models to study human brain injury and cognitive dysfunction. Mouse models are cost- and- time effective, and reproducible. Newborn mice have highly efficient redox systems compared to human preterm infants. To overcome the redox system of the newborn mice and to mimic the prolonged hyperoxic environment faced by a subset of ex-utero preterm infants in the NICU, who later develop chronic lung disease (BPD) and other co-morbidities associated with prematurity, we used a higher magnitude of oxidative stress with higher concentrations (85% O2) and a longer duration (P2–14) of oxygen as described previously^19,20^. It is possible that intermittent hyperoxia of brief duration may be a better model of the clinical situation experienced by human preterm infants as the oxygen saturation is closely monitored and maintained within a relatively narrow target range, but the effects of brief intermittent hyperoxia exposure are probably more limited and subtle, difficult to evaluate, and would require new model development (to identify frequency, duration, and magnitude of exposure). Our model, although not a perfect simulation of the human preterm infant, adequately reproduces the hippocampal shrinkage and associated memory deficit^4,44^.

In the subsequent paragraphs, we discuss our findings in the order that they are shown in the Tables.

Metabotropic glutamate receptor 7 (GLU7: Group III mGLU receptors), a presynaptic receptor, potentiates the release of glutamate^45^ and is essential for the induction of long-term potentiation (LTP) at hippocampal Schaffer collateral-CA1 synapses^46^. In this study, hyperoxia reduced hippocampal GLU7 protein levels at P14. Studies have shown that mice lacking GLU7 receptors have deficits in spatial leaning memory^47^ and exhibit reduced anxiety-related behavior^48^ and fear^49^. We have previously shown that mouse pups exposed to oxygen showed reduced fear and anxiety when assessed at adult age^19^ consistent with anticipated findings of GLU7 deficiency.

Activation of GABA_A_ and GABA_B_ subunits results in hyperpolarizing influx of chloride and potassium ions respectively. In rodent models, over-activation of GABA_A_ and GABA_B_ receptors is known to cause learning and memory deficits, especially spatial navigation deficits, and the inhibition of GABA_A_^50^ and GABA_B_^51^ improves deficits in demyelination and vascular models of dementia. Studies have shown that reactive oxygen species increase the potency of GABA_A_, increase GABA_A_ receptor-mediated tonic current in hippocampal neurons in rodent oxidative stress models^52^, and modulates GABA_A_ function^53^. Our study showed that oxidative stress increases the expression of GABA_A_ receptors, specifically α3, α5, and ɤ2 subunits in the hippocampus. The reduction in GLU7 receptor and increase in GABA_A_ receptor subunits amounts in the hippocampus noted at P14 may negatively impact initiation and/or maintenance of postsynaptic depolarization required for the induction of long-term memory in adult life.

Supraphysiological levels of oxygen exposure downregulate TrkB/ERK/AKT signaling pathways with no significant impact on the expression levels of type I and II CAMK levels. Previous studies have shown that oxidative stress decreases TrkB, and antioxidant-induced TrkB activation mitigates oxidative stress-induced neuronal apoptosis^54,55^. In this study, reduced TrkB, ERK2, PKB (AKT), mTORC1, ribosomal protein S6, and EIF4E and increased PTEN protein levels in hyperoxic mice suggest that oxidant stress adversely affects multiple pathways necessary for long-term plasticity in the developing hippocampus. The increased expression levels of immediate early genes (BDNF, ARC, EGR1, and C/EBP) in the hyperoxia-exposed group suggest hyperoxia-induced increased neuronal activity in response to oxidative stress. Neuronal hyperactivity is known to accelerate the depletion of neural stem cells and impair the hippocampal neurogenesis^56^.

Our immunoblotting for TrkB, AKT, and ERK2 at 14d and 14 weeks indicated that protein amounts were not significantly different at 14d, although they were markedly reduced by at 14 weeks in hyperoxia-exposed mice. These results are in contrast to our proteomic data, which demonstrated reductions in TrkB, AKT, and ERK2 at 14d. It is possible that the proteomic analysis was more sensitive as compared to immunoblotting, or measure different epitopes of the protein. Regardless, the marked reductions in TrkB, AKT, and ERK2 at 14 weeks by immunoblotting indicate that the abnormalities in these signaling pathways induced by oxidative stress not only persist, but may become exaggerated over time.

Our results indicate that there is oxidative DNA damage (increased 8OHdG) in the hippocampus, although not associated with significant increases in lipid peroxidation. In addition, we found reductions of proliferating cell nuclear antigen (PCNA) in the dentate gyrus, associated with increases in glial fibrillary acidic protein (GFAP) and reductions in CNPase in the CA1 region, indicating reactive astrocytosis and reduced myelin protein. Combined with our previous studies indicating reductions of hippocampal volume^19^, these current data of oxidative damage and reduced cell proliferation with reactive astrocytosis and reduced myelin demonstrate structural hippocampal injury, in addition to the alterations in signaling pathways demonstrated by proteomic and transcriptomic analyses.

The pathophysiological processes by which oxidant stress negatively affects the multiple pathways involved in long-term plasticity are likely complex. Bioinformatics analysis of over-and under-expressed proteins suggests that oxygen exposure leads to mitochondrial dysfunction and global dysfunction in protein synthesis and translation. It is probable that even proteins that are not differentially expressed have differential post-translational modifications, resulting in deficits in protein function and coordination of signaling networks. We also observed that protein changes are not consistently associated with similar changes in gene expression profiles. It is possible that feedback mechanisms or post-translational effects mediated by microRNA or protein stability (protein degradation) may be responsible for the lack of association between protein and gene expression changes. Dysfunction in mitochondrial function^57^, protein synthesis, and degradation^58,59^, are implicated in the oxidative stress associated brain disorders such as Alzheimer’s and Parkinson’s disease. Some of the findings of our model are similar to those seen following reoxygenation after profound hypoxia-ischemia in animal models^60,61^, consistent with increases in reactive oxygen species being the underlying mechanism.

Our results suggest that strategies that focus on specific signaling pathways may not be very effective as multiple signaling pathways are involved. Clinical trials with therapies such as stem cells (NCT02434965), erythropoietin (NCT03079167) and allopurinol (e.g. NCT03162653) that have more global effects are ongoing and results are anticipated shortly.

## Conclusion

Oxygen exposure alters the expression of hippocampal proteins involved in multiple signaling pathways and induces mitochondrial and global protein dysfunction. Additional mechanistic electrophysiology studies evaluating synaptic plasticity are required to determine molecular and functional correlations and underlying mechanisms by which early hyperoxia exposure leads to long-term sequelae.

## Electronic supplementary material

Supplemental Figures
