# Supplementary material for: Supraphysiological Levels of Oxygen Exposure During the Neonatal Period Impairs Signaling Pathways Required for Learning and Memory
Source: Sci Rep. 2018 Jul 2;8:9914. doi: 10.1038/s41598-018-28220-4 (PMC6028393; doi:10.1038/s41598-018-28220-4)
Supplement: Supplementary file 1 — Supplemental Figures [file 41598_2018_28220_MOESM1_ESM.doc]

Title: Supraphysiological Levels of Oxygen Exposure During the Neonatal Period Impairs Signaling Pathways Required for Learning and Memory

Abbreviated title: Oxygen-Induced Hippocampal Signaling Impairment

Author names and affiliations:

*Manimaran Ramani MD^1^, Ranjit Kumar PhD^2^, Brian Halloran MS^1^, Charitharth Vivek Lal MD^1^, Namasivayam Ambalavanan MD^1^, ^3^, and Lori L McMahon PhD^3^, ^4^

From the Departments of Pediatrics1, Bioinformatics2, Cell, Developmental, and Integrative Biology3, and Neurology4, University of Alabama at Birmingham, Birmingham, AL 35233

Corresponding author:

*Manimaran Ramani MD

University of Alabama at Birmingham, 176F Suite 9380,

619 South 20th St., Birmingham, AL 35233 USA

Tel: (205) 934-4680

Fax: (205) 934-3100

Email: [mramani@peds.uab.edu](mailto:mramani@peds.uab.edu)

**Supplemental Fig 1**: Hierarchical clustering of differentially expressed hippocampal proteins in room air vs hyperoxia exposed mice. Dendrogram above the heat map depicts hierarchical clustering of the samples (Room air shown as green, hyperoxia samples red). Cluster distance is based on the average distance between all the pairs of objects in the two clusters. Dendrograms for differentially expressed proteins are shown on the left side of the heat map. In the heat maps, red shows increased expression, blue indicates decreased expression, Expression value intensities are illustrated by color with a range of 0.5 to 3 on a log scale.


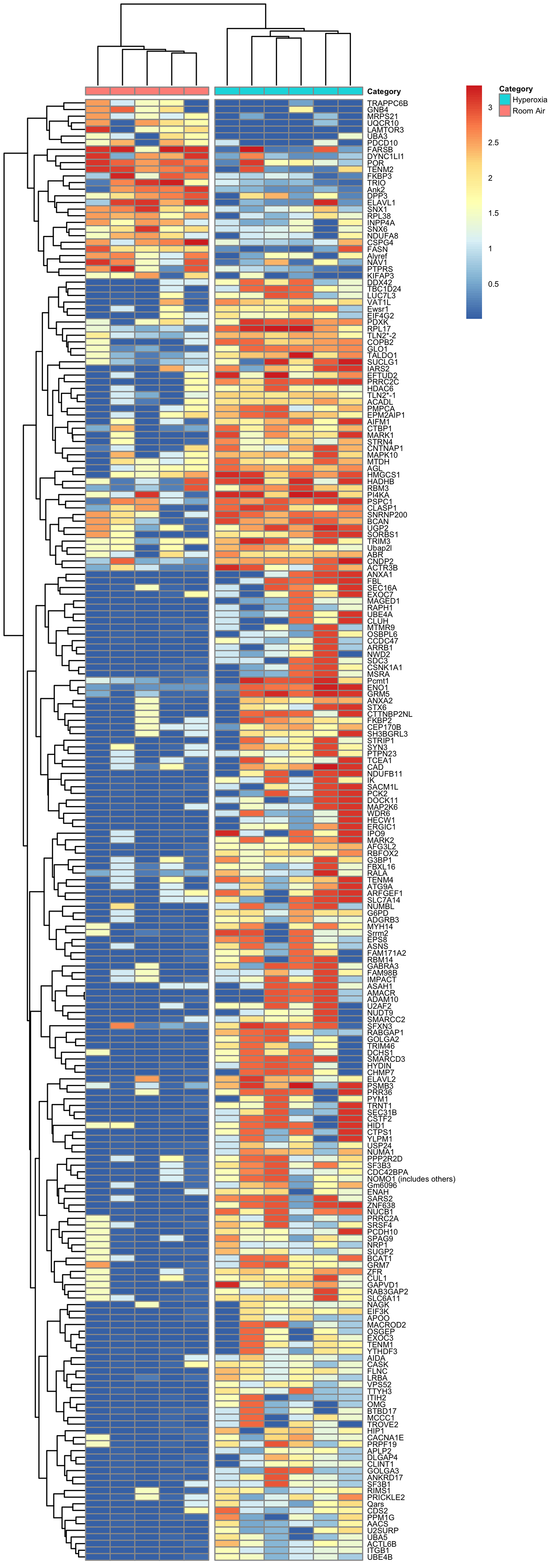


**Supplemental Fig 2:** The upstream and downstream signaling networks of AKT by proteomic analysis.


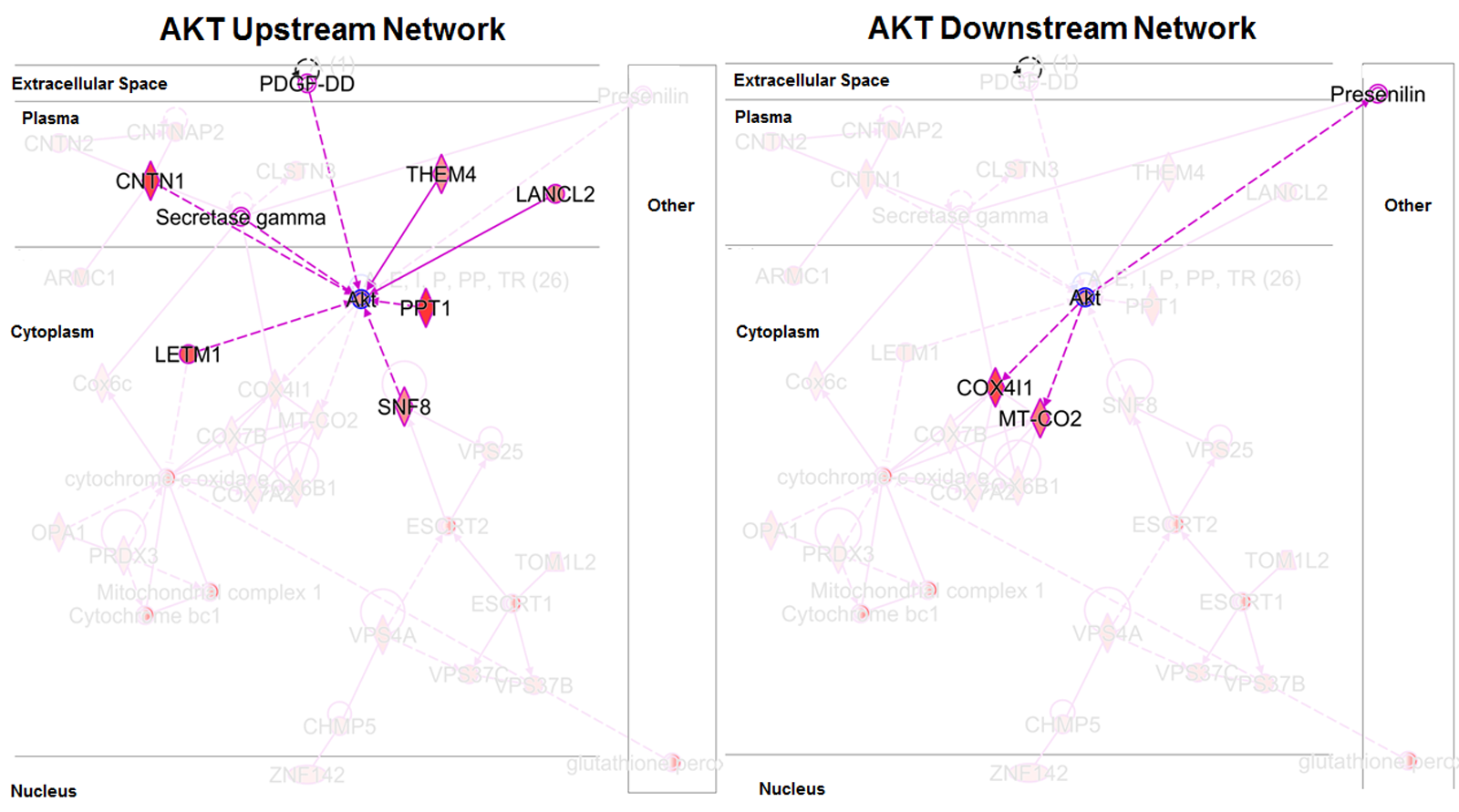


**Supplemental Fig 3:** The upstream and downstream signaling networks of ERK by proteomic analysis.


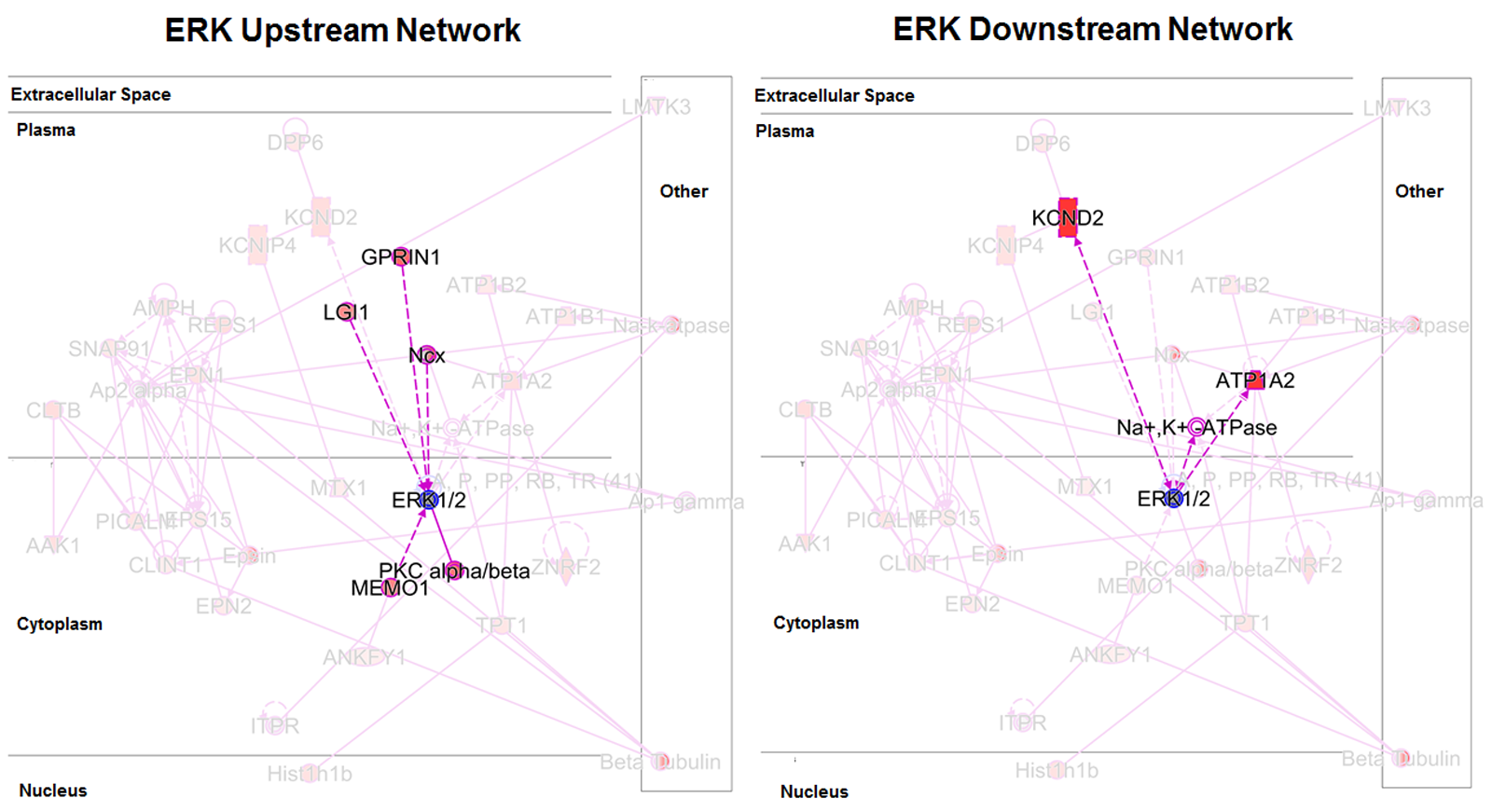


**Supplemental Table 1:** Upregulated Proteins (n=5 in Air group, 6 in Hyperoxia group) and Corresponding Gene Expression in Hyperoxia-Exposed Mice (n=3 in Air group, 3 in Hyperoxia group)

| **Symbol** | **Molecule** | **Protein**  **Log Fold Change in Hyperoxia (vs. Air)** | **P value for protein change** | **Gene Expression**  **Log Fold Change in Hyperoxia (vs. Air)** | **P value for gene expression change** |
| --- | --- | --- | --- | --- | --- |
| PRRC2C | Proline Rich Coiled-Coil 2C | +2.42 | 0.0008 | -0.34 | **0.047** |
| NUCB1 | Nucleobindin 1 | +2.32 | 0.0006 | +**0.25** | **0.04** |
| ZNF638 | Zinc finger protein 638 | +2.32 | 0.003 | -0.13 | 0.41 |
| GRM5 | Glutamate metabotropic receptor 5 | +2.25 | 0.004 | +0.09 | 0.09 |
| MARK2 | Microtubule affinity regulating kinase 2 | +2.20 | 0.0001 | +0.18 | **0.04** |
| SMARCD3 | SWI/SNF related, matrix associated, actin dependent | +2.19 | 0.003 | -0.08 | 0.56 |
| FBL | Fibrillarin | +2.16 | 0.009 | +0.27 | **0.04** |
| ENO1 | Enolase 1 | +2.15 | 0.004 | +0.06 | 0.74 |
| MARK1 | Microtubule affinity regulating kinase 1 | +2.06 | 0.001 | **-0.29** | **0.004** |
| SF3B3 | Splicing factor 3b subunit 3 | +2.02 | 0.0001 | -0.02 | 0.91 |
| RBFOX2 | RNA binding protein, fox-1 homolog 2 | +2.01 | 0.005 | -**0.26** | **0.004** |
| RPL17 | Ribosomal protein L17 | +2.01 | 0.0001 | +**0.34** | **0.01** |
| COPB2 | Coatomer protein complex subunit beta 2 | +2.01 | 0.002 | +0.15 | 0.13 |
| SARS2 | Seryl-tRNA synthetase 2, mitochondria | +1.97 | 0.003 | +0.19 | 0.32 |
| GLO1 | Glyoxalase 1 | +1.97 | 0.0003 | +0.09 | 0.54 |
| CTPS1 | CTP synthase 1 | +1.94 | 0.008 | -0.09 | 0.58 |
| IK | IK cytokine, down-regulator of HLA II | +1.90 | 0.002 | +**0.54** | **4.53E-07** |
| TBC1D24 | TBC1 domain family member 24 | +1.89 | 0.001 | -0.18 | 0.13 |
| PRR36 | Proline rich 36 | +1.89 | 0.01 | +0.31 | 0.06 |
| TRNT1 | tRNA nucleotidyl transferase 1 | +1.89 | 0.004 | -0.07 | 0.72 |
| PSMB3 | Proteasome subunit beta 3 | +1.89 | 0.003 | -0.21 | 0.20 |
| NUMA1 | Nuclear mitotic apparatus protein 1 | +1.88 | 0.002 | +0.18 | 0.15 |
| RABGAP1 | RAB GTPase activating protein 1 | +1.87 | 0.005 | -0.11 | 0.27 |
| CTTNBP2NL | CTTNBP2 N-terminal like | +1.85 | 0.013 | **-0.34** | **0.02** |
| GAPVD1 | GTPase activating protein and VPS9 domains 1 | +1.84 | 0.001 | **-0.30** | **0.0001** |
| AFG3L2 | AFG3 like matrix AAA peptidase subunit 2 | +1.83 | 0.006 | -0.03 | 0.82 |
| NDUFB11 | NADH:ubiquinone oxidoreductase subunit B11 | +1.81 | 0.03 | +0.13 | 0.36 |
| PCMT1 | Protein l-isoaspartate (D-aspartate) O-methyltransferase 1 | +1.81 | 0.013 | **-0.26** | **0.01** |
| FLNC | Filamin C | +1.80 | 0.001 | **-0.50** | **0.04** |
| CSTF2 | Cleavage stimulation factor subunit 2 | +1.80 | 0.01 | **-0.41** | **0.00001** |
| GABRA3 | Gamma aminobutyric acid type A receptor alpha 3 subunit | +1.80 | 0.007 | **-0.85** | **1.24E-06** |
| HYDIN | HYDIN, axonemal central pair apparatus protein | +1.79 | 0.01 | +0.15 | 0.62 |
| EFTUD2 | Elongation factor Tu GFP binding domain containing 2 | +1.78 | 0.005 | +0.19 | 0.07 |
| G6PD | Glucose-6-phosphate dehydrogenase | +1.77 | 0.0001 | +0.05 | 0.78 |
| PCK2 | phosphoenolpyruvate carboxykinase 2, mitochondrial | +1.74 | 0.02 | +0.09 | 0.64 |
| AIFM1 | Apoptosis inducing factor mitochondria associated 1 | +1.74 | 0.003 | -0.16 | 0.22 |
| IPO9 | Importin 9 | +1.73 | 0.02 | **-0.30** | **0.0001** |
| UBE4A | Ubiquitination factor E4A | +1.71 | 0.01 | **-0.30** | **0.008** |
| SEC31B | SEC31 homolog B, COPII coat complex component | +1.70 | 0.01 | +0.13 | 0.68 |
| EPS8 | Epidermal growth factor receptor pathway substrate 8 | +1.69 | 0.01 | -0.14 | 0.30 |
| STRIP1 | Striatin interacting protein 1 | +1.69 | 0.01 | +0.06 | 0.63 |
| ANXA1 | Annexin A1 | +1.69 | 0.03 | **-0.65** | **0.02** |
| GOLGA2 | Golgin A2 | +1.69 | 0.01 | +**0.20** | **0.04** |
| RBM14 | RNA binding motif protein 14 | +1.68 | 0.03 | +0.22 | 0.08 |
| CHMP7 | Charged multivesicular body protein 7 | +1.68 | 0.03 | -0.06 | 0.62 |
| ELAVL2 | ELAV like RNA binding protein 2 | +1.68 | 0.02 | -0.12 | 0.57 |
| SACM1L | SAC1 suppressor of actin mutations 1 like (yeast) | +1.66 | 0.02 | **-0.39** | **0.04** |
| TALDO1 | Transaldolase 1 | +1.66 | 0.0005 | -0.07 | 0.72 |
| TENM4 | Teneurin transmembrance protein 4 | +1.65 | 0.009 | **-0.36** | **0.01** |
| CAD | carbamoyl-phosphate synthetase 2, aspartate transcarbamylase, and dihydroorotase | +1.65 | 0.02 | -0.19 | 0.17 |
| STRN4 | Striatin 4 | +1.64 | 0.003 | +0.04 | 0.75 |
| CDC42BPA | CDC42 binding protein kinase alpha | +1.64 | 0.001 | -0.11 | 0.46 |
| PMPCA | Peptidase mitochondrial processing alpha subunit | +1.63 | 0.006 | **-0.25** | **0.01** |
| BCAT1 | Branched chain amino acid transaminase 1 | +1.63 | 0.005 | -0.09 | 0.37 |
| VAT1L | Vesicle amine transport 1 like | +1.62 | 0.025 | **-0.87** | **2.67E-07** |
| TENM1 | Teneurin transmembrane protein 1 | +1.61 | 0.009 | **-0.78** | **0.0001** |
| HIP1 | Huntingtin interacting protein 1 | +1.61 | 0.007 | **-0.59** | **2.97E-08** |
| AMACR | Alpha-methylacyl-CoA racemase | +1.59 | 0.04 | +0.31 | 0.07 |
| CNDP2 | Carnosine dipeptidase 2 | +1.58 | 0.02 | +0.003 | 0.98 |
| ASNS | Asparagine synthetase | +1.58 | 0.007 | +0.33 | 0.02 |
| ZFR | Zinc finger RNA binding protein | +1.58 | 0.004 | -0.17 | 0.20 |
| NOMO1 | NODAL modulator 1 | +1.58 | 0.006 | -0.14 | 0.15 |
| HID1 | HID1 domain containing | +1.57 | 0.03 | +0.20 | 0.08 |
| EIF3K | Eukaryotic translation initiation factor 3 subunit K | +1.57 | 0.005 | +0.28 | 0.04 |
| ARFGEF1 | ADP ribosylation factor guanine nucleotide exchange factor 1 | +1.57 | 0.02 | **-0.34** | **0.02** |
| ACADL | Acyl-CoA dehydrogenase, long chain | +1.57 | 0.008 | +0.09 | 0.61 |
| SEC16A | SEC16 homolog A, endoplasmic reticulum export factor | +1.56 | 0.02 | -0.18 | 0.17 |
| BCAN | Brevican | +1.56 | 0.03 | -0.07 | 0.72 |
| SORBS1 | Sorbin and SH3 domain containing 1 | +1.56 | 0.03 | -0.27 | 0.20 |
| ADAM10 | ADAM metallopeptidase domain 10 | +1.56 | 0.04 | -0.08 | 0.60 |
| WDR6 | WD repeat domain 6 | +1.56 | 0.02 | +**0.37** | **0.0007** |
| YLPM1 | YLP motif containing 1 | +1.55 | 0.02 | -0.08 | 0.71 |
| ERGIC1 | Endoplasmic reticulum-golgi intermediate compartment 1 | +1.54 | 0.02 | +0.11 | 0.44 |
| SLC6A11 | Solute carrier family 6 member 11 | +1.53 | 0.004 | -0.27 | 0.10 |
| U2AF2 | U2 small nuclear RNA auxillary factor 2 | +1.53 | 0.01 | +**0.24** | **0.02** |
| CNTNAP1 | Contactin associated protein 1 | +1.53 | 0.01 | +**0.68** | **3.03E-11** |
| CSNK1A1 | Casein kinase 1 alpha 1 | +1.53 | 0.02 | +0.03 | 0.85 |
| SDC3 | Syndecan 3 | +1.53 | 0.02 | **-0.29** | **0.04** |
| ANXA2 | Annexin A2 | +1.53 | 0.01 | -0.33 | 0.10 |
| SFXN3 | Sideroflexin 3 | +1.53 | 0.04 | +0.20 | 0.14 |
| MYH14 | Myosin heavy chain 14f | +1.52 | 0.01 | +0.19 | 0.25 |
| DOCK11 | Dedicator of cytokinesis 11 | +1.52 | 0.03 | +0.18 | 0.42 |
| ANKRD17 | Ankyrin repeat domain 17 | +1.52 | 0.004 | **-0.30** | **0.03** |
| IARS2 | Isoleucyl-tRNA synthetase 2, mitochondrial | +1.52 | 0.03 | -0.02 | 0.86 |
| ACTR3B | ARP3 actin related protein 3 homolog B | +1.52 | 0.01 | -0.11 | 0.37 |
| SRRM2 | Serine/arginine repetitive matrix 2 | +1.51 | 0.03 | +0.22 | 0.27 |
| STX6 | Syntaxin 6 | +1.51 | 0.02 | +**0.26** | **0.001** |
| CTBP1 | C-terminal binding protein 1 | +1.50 | 0.01 | -0.15 | 0.25 |
| TLN2 | Talin 2 | +1.50 | 0.002 | **-0.46** | **0.00001** |
| TRIM46 | Tripartite motif containing 46 | +1.49 | 0.01 | +**0.28** | **0.01** |
| TTYH3 | Tweety family member 3 | +1.49 | 0.005 | +0.05 | 0.78 |
| CLASP1 | Cytoplasmic linker associated protein 1 | +1.49 | 0.04 | -0.11 | 0.37 |
| G3BP1 | G3BP stress granule assembly factor 1 | +1.48 | 0.008 | -0.15 | 0.14 |
| LRBA | LPS responsive beige-like anchor protein | +1.47 | 0.001 | -0.01 | 0.96 |
| PI4KA | Phosphatidylinositol 4-kinase alpha | +1.47 | 0.03 | +0.004 | 0.98 |
| SLC7A14 | Solute carrier family member 14 | +1.47 | 0.04 | +0.20 | 0.34 |
| PRPF19 | Pre-mRNA processing factor 19 | +1.46 | 0.009 | +0.08 | 0.47 |
| USP24 | Ubiquitin specific peptidase 24 | +1.46 | 0.002 | **-0.35** | **0.001** |
| FAM171A2 | Family with sequence similarity 171 member A2 | +1.45 | 0.01 | -0.09 | 0.57 |
| RBM3 | RNA binding motif (RNP1, RRM) protein 3 | +1.45 | 0.04 | +**0.56** | **0.00007** |
| LUC7L3 | LUC7 like 3 pre-mRNA splicing factor | +1.45 | 0.01 | +0.34 | 0.07 |
| PCDH10 | Protocadherin 10 | +1.45 | 0.008 | **-0.59** | **0.0008** |
| PSPC1 | Paraspeckle component 1 | +1.44 | 0.04 | +0.04 | 0.78 |
| EPM2AIP1 | EPM2A interacting protein 1 | +1.44 | 0.02 | **-0.56** | **5.63E-06** |
| HADHB | Hydroxyacyl-CoA dehydrogenase/3-ketoacyl-CoA | +1.44 | 0.04 | -0.13 | 0.48 |
| NUMBL | NUMB like, endocytic adaptor protein | +1.43 | 0.04 | +**0.39** | **0.002** |
| DCHS1 | Dachsous cadherin related 1 | +1.43 | 0.02 | **-0.32** | **0.01** |
| DDX42 | DEAD-box helicase 42 | +1.43 | 0.009 | +0.11 | 0.38 |
| ASAH1 | N-acylsphingosine amidohydrolase 1 | +1.43 | 0.03 | **-0.26** | **0.02** |
| CLUH | Clustered mitochondria homolog | +1.42 | 0.04 | +0.04 | 0.73 |
| GOLGA3 | Golgin A3 | +1.42 | 0.02 | -0.05 | 0.76 |
| CCDC47 | Coiled-coil domain containing 47 | +1.42 | 0.01 | +0.06 | 0.68 |
| VPS52 | VPS52, GARP complex subunit | +1.42 | 0.0006 | +0.16 | 0.13 |
| Ewsr1 | Ewing sarcoma breakpoint region 1 | +1.41 | 0.01 | +**0.45** | **0.0002** |
| FKBP2 | FK506 binding protein 2 | +1.41 | 0.02 | +**0.76** | **7.26E-11** |
| MAP2K6 | Mitogen activated protein kinase 6 | +1.41 | 0.03 | +0.10 | 0.58 |
| GRM7 | Glutamate metabotropic receptor 7 | +1.41 | 0.04 | -0.03 | 0.89 |
| ACTL6B | Actin like 6B | +1.41 | 0.001 | +**0.50** | **0.00001** |
| EXOC7 | Exocyst complex component 7 | +1.40 | 0.03 | +**0.23** | **0.02** |
| PPP2R2D | Protein phosphatase 2 regulatory subunit Bdelta | +1.40 | 0.01 | +0.11 | 0.35 |
| MACROD2 | MACRO domain containing 2 | +1.38 | 0.03 | **-0.23** | **0.04** |
| ATG9A | Autophagy related 9A | +1.37 | 0.01 | -0.09 | 0.46 |
| PDXK | Pyridoxal kinase | +1.37 | 0.01 | -0.27 | 0.16 |
| MCCC1 | Methylcrotonoyl-CoA carboxylase I | +1.37 | 0.01 | -0.24 | 0.14 |
| RAB3GAP2 | RAB3 GTPase activating non-catalytic protein subunit 2 | +1.36 | 0.009 | **-0.39** | **0.0006** |
| PYM1 | PYM homolog 1, exon junction complex associated factor | +1.36 | 0.03 | +0.22 | 0.15 |
| HDAC6 | Histone deactylase 6 | +1.36 | 0.01 | +0.21 | 0.11 |
| SUCLG1 | Succinate-CoA ligase alpha subunit | +1.36 | 0.03 | +0.08 | 0.62 |
| YTHDF3 | YTH N6-methyladenosine RNA binding protein 3 | +1.36 | 0.02 | **-0.35** | **0.0007** |
| AGL | Amylo-alpha-1, 6-glucosidase, 4-alpha-glucanotransferase | +1.35 | 0.01 | -0.21 | 0.16 |
| CUL1 | Cullin 1 | +1.35 | 0.02 | -0.17 | 0.09 |
| MSRA | Methionine sulfoxide reductase A | +1.34 | 0.04 | +**0.34** | **0.01** |
| SMARCC2 | SWI/SNF related, matrix associated, actin dependent regulator of chromatin subfamily c member 2 | +1.34 | 0.02 | +**0.33** | **0.001** |
| UGP2 | SURP and G-patch domain containing 2 | +1.32 | 0.03 | -0.02 | 0.92 |
| SYN3 | Synapsin III | +1.32 | 0.03 | **-0.42** | **0.04** |
| ARRB1 | Arrestin beta 1 | +1.31 | 0.02 | **-0.44** | **0.0004** |
| IMPACT | Impact RWD domain protein | +1.30 | 0.02 | -0.22 | 0.06 |
| ADGRB3 | Adhesion G protein-coupled receptor B3 | +1.30 | 0.009 | +0.02 | 0.92 |
| MAPK10 | Mitogen-activated protein kinase 10 | +1.30 | 0.04 | +**0.27** | **0.01** |
| EXOC3 | Exocyst complex component 3 | +1.29 | 0.03 | -0.32 | 0.31 |
| PTPN23 | Protein tyrosine phosphatase, non-receptor type 23 | +1.29 | 0.009 | +**0.37** | **0.004** |
| TCEA1 | Transcription elongation factor A1 | +1.28 | 0.04 | -0.11 | 0.41 |
| ENAH | Enabled homolog (Drosophila) | +1.28 | 0.01 | **-0.30** | **0.01** |
| SNRNP200 | Small nuclear ribonucleoprotein U5 subunit 200 | +1.27 | 0.04 | **-0.30** | **0.01** |
| NWD2 | NACHT and WD repeat domain containing 2 | +1.25 | 0.03 | **-0.42** | **0.02** |
| HMGCS1 | 3-hydroxy-3-methylglutaryl-CoA synthase 1 | +1.24 | 0.03 | -0.08 | 0.64 |
| CACNA1E | Calcium voltage-gated channel subunit alpha1 E | +1.24 | 0.01 | +0.05 | 0.84 |
| SH3BGRL3 | SH3 domain binding glutamate rich protein like 3 | +1.24 | 0.04 | +**0.55** | **0.00001** |
| SRSF4 | Serine and arginine rich splicing factor 4 | +1.23 | 0.02 | +0.02 | 0.93 |
| OSBPL6 | Oxysterol binding protein like 6 | +1.23 | 0.03 | -0.10 | 0.63 |
| FAM98B | Family with sequence similarity 98 member B | +1.21 | 0.03 | +**0.40** | **0.0005** |
| GLNRS | Glutaminyl-tRNA-synthetase | +1.21 | 0.006 | -0.39 | 0.17 |
| SF3B1 | Splicing factor 3b subunit 1 | +1.21 | 0.01 | -0.18 | 0.28 |
| HECW1 | HECT, C2, and WW domain containing E3 ubiquitin protein ligase 1 | +1.20 | 0.03 | **-0.65** | **7.54E-07** |
| NUDT9 | Nudix hydrolase 9 | +1.20 | 0.04 | +**0.40** | **0.00005** |
| DLGAP4 | DLG associated protein 4 | +1.19 | 0.01 | +0.16 | 0.19 |
| TROVE2 | TROVE domain family member 2 | +1.18 | 0.04 | **-0.60** | **0.00009** |
| PPM1G | Protein-phosphatase, Mg2+/Mn2+ dependent 1G | +1.18 | 0.03 | +**0.26** | **0.02** |
| CDS2 | CDP-diacylglycerol synthase 2 | +1.17 | 0.03 | **-0.31** | **0.001** |
| ITGB1 | Integrin subunit beta 1 | +1.17 | 0.004 | **-0.46** | **0.001** |
| APLP2 | Amyloid beta precursor like protein 2 | +1.17 | 0.001 | -0.07 | 0.5 |
| RIMS1 | Regulating synaptic membrane exocytosis 1 | +1.17 | 0.01 | -0.24 | 0.19 |
| CLINT1 | Clathrin interactor 1 | +1.16 | 0.005 | -0.02 | 0.87 |
| BTBD17 | BTB domain containing 17 | +1.16 | 0.03 | -0.31 | 0.07 |
| OSGEP | 0-sialoglycoprotein endopeptidase | +1.15 | 0.04 | +0.19 | 0.08 |
| OMG | Oligodendrocyte myelin glycoprotein | +1.15 | 0.03 | +**0.28** | **0.02** |
| TLN2 | Talin 2 | +1.14 | 0.002 | **-0.46** | **0.00001** |
| SUGP2 | SURP and G-patch domain containing 2 | +1.13 | 0.017 | +0.02 | 0.91 |
| MTMR9 | Mytotubulin related protein 9 | +1.13 | 0.04 | **-0.77** | **4.36E-06** |
| RALA | RAS like proto-oncogene A | +1.12 | 0.02 | -0.05 | 0.66 |
| AIDA | Axin interactor, dorsalization associated | +1.12 | 0.005 | -0.11 | 0.39 |
| UBAP2L | Ubiquitin-associated protein 2 like | +1.12 | 0.04 | +**0.46** | **0.0006** |
| UBE4B | Ubiquitination factor E4B | +1.12 | 0.008 | -0.30 | 0.35 |
| APOO | Apolipoprotein O | +1.11 | 0.02 | +0.12 | 0.74 |
| CEP170B | Centrosomal protein 170B | +1.11 | 0.03 | **-0.46** | **0.0006** |
| PRRC2A | Proline rich coiled-coil 2A | +1.11 | 0.04 | +**0.27** | **0.01** |
| AACS | Acetoacetyl-CoA-synthetase | +1.10 | 0.008 | **-0.30** | **0.0004** |
| MAGED1 | MAGE family member D1 | +1.10 | 0.03 | +0.14 | 0.23 |
| U2SURP | U2 snRNP associated SURP domain containing | +1.09 | 0.02 | **-0.26** | **0.02** |
| RAPH1 | Ras association (RalGDS/AF-6) and pleckstrin homology | +1.09 | 0.03 | **-0.42** | **0.001** |
| ABR | Active BCR-related | +1.06 | 0.03 | **-0.93** | **1.18E-08** |
| SPAG9 | Sperm associated antigen 9 | +1.06 | 0.03 | **-0.41** | **0.003** |
| CASK | Calcium/calmodulin dependent serine protein kinase | +1.06 | 0.03 | **-0.49** | **0.00004** |
| EIF4G2 | Eukaryotic translation initiator factor 4 gamma 2 | +1.05 | 0.03 | **-0.31** | **0.003** |
| ITIH2 | Inter-alpha-trypsin inhibitor heavy chain 2 | +1.05 | 0.04 | -0.33 | 0.23 |
| PRICKLE2 | Prickle planar cell polarity protein 2 | +1.05 | 0.04 | +0.12 | 0.47 |
| NRP1 | Neuropilin 1 | +1.04 | 0.02 | +0.06 | 0.74 |
| TRIM3 | Tripartite motif containing 33 | +1.04 | 0.37 | **-0.32** | **0.0001** |
| FBXL16 | F-box and Leucine rich repeat protein 16 | +1.03 | 0.03 | +**0.49** | **0.0001** |
| MTDH | Metadherin | +1.02 | 0.04 | **-0.31** | **0.04** |
| UBA5 | Ubiquitin like modifier activating enzyme 5 | +1.00 | 0.01 | +**0.69** | **0.0005** |
| NAGK | n-acetylglucosamine kinase | +1.00 | 0.04 | +0.10 | 0.54 |

**Supplemental Table 2:** Downregulated Proteins (n=5 in Air group, 6 in Hyperoxia group) and Corresponding Gene Expression in Hyperoxia-Exposed Mice (n=3 in Air group, 3 in Hyperoxia group)

| **Symbol** | **Molecule** | **Protein**  **Log Fold Change in Hyperoxia (vs. Air)** | **P value for protein change** | **Gene Expression**  **Log Fold Change in Hyperoxia (vs. Air)** | **P value for gene expression change** |
| --- | --- | --- | --- | --- | --- |
| TENM2 | Teneurin transmembrane protein 2 | -1.86 | 0.01 | -0.27 | 0.15 |
| Ank2 | Ankyrin 2, brain | -1.75 | 0.03 | **-0.31** | **0.01** |
| LAMTOR3 | Late endosomal/lysosomal adaptor, MAPK and MTOR activator 3 | -1.69 | 0.02 | -0.04 | 0.79 |
| ELAVL1 | ELAV like RNA binding protein 1 | -1.68 | 0.02 | -0.21 | 0.06 |
| UQCR10 | Ubiquinol-cytochrome c reductase, complex III subunit X | -1.67 | 0.01 | -0.25 | 0.15 |
| FARSB | Phenylalanyl-tRNA synthetase beta subunit | -1.64 | 0.04 | -0.11 | 0.40 |
| TRIO | Trio Rho guanine nucleotide exchange factor | -1.63 | 0.04 | -0.10 | 0.56 |
| FASN | Fatty acid synthase | -1.57 | 0.01 | **-0.23** | **0.03** |
| GNB4 | G protein subunit beta 4 | -1.50 | 0.03 | **-0.36** | **0.004** |
| PTPRS | Protein tyrosine phosphatase, receptor type S | -1.49 | 0.04 | +0.08 | 0.63 |
| FKBP3 | FK506 binding protein 3 | -1.46 | 0.03 | +0.11 | 0.31 |
| DYNC1LI1 | Dynein cytoplasmic 1 light intermediate chain 1 | -1.44 | 0.04 | **-0.24** | **0.01** |
| MRPS21 | Mitochondrial ribosomal protein S21 | -1.38 | 0.003 | **+0.29** | **0.01** |
| mGLU7 | Glutamate Receptor, Metabotropic 7 | -1.44 | 0.01 | -0.03 | 0.89 |
| POR | Cytochrome p450 oxidoreductase | -1.34 | 0.01 | **+0.40** | **0.0003** |
| ERK2 | Extracellular Signal-Regulated Kinase 2 | -1.33 | 0.02 | +0.16 | 0.10 |
| NAV1 | Neuron navigator 1 | -1.32 | 0.03 | **-0.33** | **0.02** |
| RPL38 | Ribosomal protein L38 | -1.28 | 0.01 | **-0.44** | **0.02** |
| TRAPPC6B | Trafficking protein particle complex 6B | -1.28 | 0.03 | **+0.33** | **0.0008** |
| KIFAP3 | Kinesin associated protein 3 | -1.26 | 0.03 | -0.18 | 0.17 |
| PDCD10 | Programmed cell death 10 | -1.22 | 0.04 | +0.25 | 0.02 |
| ALYREF | Aly/REF export factor | -1.20 | 0.03 | +0.16 | 0.26 |
| SNX1 | Sorting nexin 1 | -1.20 | 0.007 | +0.21 | 0.08 |
| CSPG4 | Chondroitin sulfate proteoglycan 4 | -1.20 | 0.02 | **-0.37** | **0.02** |
| EIF4E | Eukaryotic translation initiation factor 4e | -1.18 | 0.008 | **+0.34** | **0.04** |
| DPP3 | Dipeptidyl peptidase 3 | -1.18 | 0.04 | **-0.37** | **0.004** |
| INPP4A | Inositol polyphosphate-4-phosphatase type I A | -1.15 | 0.01 | -0.15 | 0.12 |
| TrkB | Tropomyosin receptor kinase B | -1.09 | 0.04 | +0.01 | 0.96 |
| NDUFA8 | NADH:ubiquinone oxidoreductase subunit 8 | -1.07 | 0.02 | +0.22 | 0.15 |
| SNX6 | Sorting nexin 6 | -1.07 | 0.01 | -0.08 | 0.55 |
| UBA3 | Ubiquitin like modifier activating enzyme | -1.04 | 0.04 | **+0.19** | **0.05** |
| PKB-Akt3 | Protein kinase B | -1.03 | 0.02 | **-0.71** | **1.52E-07** |

**Supplemental Table 3:** Upregulated Genes in Hyperoxia-Exposed Mice (n=3/Group, P= Hyperoxia vs. Air group)

| **Symbol** | **Molecule** | **Gene Expression**  **Log Fold Change in Hyperoxia (vs. Air)** | **P value for gene expression change** |
| --- | --- | --- | --- |
| ARC | Activity-regulated cytoskeleton-associated protein | +1.51 | 5.1E-36 |
| PLA2G2F | Group IIF secretory phospholipase A2 | +1.25 | 9.09E-09 |
| HTRA4 | Serine protease HTRA4 | +1.21 | 2.81E-08 |
| NDUFA1 | NADH dehydrogenase [ubiquinone] 1 alpha subcomplex subunit 1 | +1.18 | 1.53E-18 |
| DDT | D-dopachrome decarboxylase | +1.17 | 8.82E-13 |
| PRRT1 | Proline-rich transmembrane protein 1 | +1.15 | 5.33E-14 |
| CARNS1 | Carnosine synthase 1 | +1.15 | 7.62E-16 |
| DNASE1L2 | Deoxyribonuclease-1-like 2 | +1.15 | 2.9E-12 |
| SNCG | Gamma-synuclein | +1.12 | 1.03E-07 |
| ESPN | Espin | +1.12 | 2.26E-08 |
| MAL | Myelin and lymphocyte protein | +1.09 | 4.37E-07 |
| KCNC3 | Potassium voltage-gated channel subfamily C member 3 | +1.08 | 5.29E-12 |
| TM6SF2 | Transmembrane 6 superfamily member 2 | +1.06 | 2.11E-07 |
| KLK10 | Kallikrein j | +1.06 | 9.92E-07 |
| KRT9 | Keratin, type I cytoskeletal 9 | +1.05 | 2.4E-11 |
| GRASP | General receptor for phosphoinositides 1-associated scaffold protein | +1.05 | 2.78E-20 |
| CHGB | Chromogranin B | +1.02 | 9.14E-21 |
| SLIRP | SRA stem-loop-interacting RNA-binding protein | +1.02 | 1.53E-10 |
| ATP6V0C | V-type proton ATPase 16 kDa proteolipid subunit | +1.02 | 3.95E-21 |
| TNFRSF25 | Tumor necrosis factor receptor superfamily, member 25 | +1.00 | 8.02E-06 |

**Supplemental Table 4:** Downregulated Genes in Hyperoxia-Exposed Mice (n=3/Group, P= Hyperoxia vs. Air group)

| **Symbol** | **Name** | **Log Fold Change in Hyperoxia Group** | **P value** |
| --- | --- | --- | --- |
| APOLD1 | Apolipoprotein L domain-containing 1 | -2.08 | 6.62E-39 |
| COL24A1 | Collagen alpha-1(XXIV) chain | -1.79 | 1.274E-22 |
| BGLAP3 | Osteocalcin-related protein | -1.67 | 3.29E-14 |
| LGR6 | Leucine-rich repeat-containing G-protein coupled receptor 6 | -1.56 | 1.32E-31 |
| DGKK | Diacylglycerol kinase | -1.56 | 6.06E-14 |
| FZD3 | Frizzled-3 | -1.49 | 4.74E-14 |
| IER3 | Immediate Early Response 3 | -1.48 | 4.58E-19 |
| KCNA3 | Potassium voltage-gated channel subfamily A member 3 | -1.47 | 3.73E-11 |
| VGLUT2 | Vesicular glutamate transporter 2 | -1.45 | 6.73E-19 |
| KCNK9 | Potassium channel subfamily K member 9 | -1.40 | 1.27E-11 |
| PCDH11X | Protocadherin 11 X-linked | -1.39 | 1.30E-17 |
| ANKRD34C | Ankyrin repeat domain-containing protein 34C | -1.39 | 1.79E-10 |
| USP43 | Ubiquitin carboxyl-terminal hydrolase 43 | -1.37 | 2.15E-12 |
| EDN1 | Endothelin-1 | -1.35 | 9.30E-14 |
| SLC22A3 | Solute carrier family 22 member 3 | -1.35 | 2.57E-11 |
| FOXP2 | Forkhead box protein P2 | -1.34 | 2.82E-11 |
| GM9938 | Putative uncharacterized protein | -1.32 | 3.60E-10 |
| TSHZ3 | Teashirt homolog 3 | -1.30 | 5.447E-09 |
| TSHZ2 | Teashirt homolog 2 | -1.26 | 8.062E-09 |
| RXFP1 | Relaxin receptor 1 | -1.25 | 1.14E-09 |
| UNC5D | Netrin receptor UNC5D | -1.23 | 6.17E-14 |
| LCOR | Ligand-dependent corepressor | -1.23 | 3.40E-08 |
| ADCYAP1 | Pituitary adenylate cyclase-activating polypeptide | -1.21 | 9.58E-12 |
| SLC9A7 | Sodium/hydrogen exchanger 7 | -1.20 | 3.71E-09 |
| XKR4 | XK-related protein 4 | -1.19 | 3.61E-08 |
| NPSR1 | Neuropeptide S receptor | -1.19 | 6.14E-08 |
| GPRIN3 | G protein-regulated inducer of neurite outgrowth 3 | -1.17 | 2.67E-12 |
| TOX | Thymocyte selection-associated high mobility group box protein TOX | -1.17 | 5.51E-13 |
| GPR21 | Probable G-protein coupled receptor 21 | -1.17 | 7.19E-08 |
| RN18S-RS5 | E3 ubiquitin-protein ligase RNF152 | -1.16 | 1.89E-07 |
| PRPH | Peripherin | -1.15 | 1.41E-10 |
| ZBTB37 | Putative uncharacterized protein | -1.15 | 4.34E-10 |
| FNBP1L | Formin-binding protein 1-like | -1.15 | 4.02E-16 |
| ANKFN1 | Ankyrin-repeat and fibronectin type III domain-containing 1 | -1.15 | 1.69E-07 |
| CDH7 | Cadherin-7 | -1.14 | 1.92E-09 |
| KLHL28 | Kelch-like protein 28 | -1.11 | 2.10E-09 |
| CHML | Rab proteins geranylgeranyltransferase component A 2 | -1.11 | 2.79E-13 |
| ADAMTS16 | A disintegrin and metalloproteinase with thrombospondin motifs 16 | -1.11 | 4.52E-12 |
| NREP | Neuronal regeneration-related protein | -1.11 | 7.54E-13 |
| SEMA3F | Semaphorin-3F | -1.10 | 6.81E-09 |
| MMRN1 | Multimerin-1 | -1.09 | 1.11E-06 |
| KCNH5 | Potassium voltage-gated channel subfamily H member 5 | -1.09 | 5.54E-08 |
| OPRK1 | Kappa-type opioid receptor | -1.09 | 1.16E-07 |
| WDFY4 | WD repeat and FYVE domain-containing 4 | -1.08 | 4.58E-09 |
| LAMC2 | Laminin subunit gamma-2 | -1.06 | 1.43E-06 |
| PTGFRN | Prostaglandin F2 receptor negative regulator | -1.06 | 9.97E-15 |
| AHNAK2 | AHNAK nucleoprotein 2 | -1.05 | 2.72E-07 |
| ADAM33 | Disintegrin and metalloproteinase domain-containing protein 33 | -1.05 | 4.60E-07 |
| TRIM66 | Tripartite motif-containing protein 66 | -1.04 | 1.61E-10 |
| DSC2 | Desmocollin-2 | -1.04 | 3.66E-06 |
| FABP7 | Fatty acid-binding protein, brain | -1.04 | 3.22E-10 |
| ADRA1A | Alpha-1A adrenergic receptor | -1.03 | 1.86E-08 |
| EFNA5 | Ephrin-A5 | -1.03 | 3.56E-06 |
| GABRQ | Gamma-aminobutyric acid receptor subunit theta | -1.03 | 2.35E-06 |
| ZKSCAN16 | Zinc finger with KRAB and SCAN domains 16 | -1.03 | 9.72E-08 |
| TET1 | Methylcytosine dioxygenase TET1 | -1.02 | 2.87E-12 |
| CBLN2 | Cerebellin-2 | -1.01 | 7.81E-10 |
| KLHDC8A | Kelch domain-containing protein 8A | -1.01 | 3.82E-08 |
| PAPLN | Papilin | -1.00 | 3.21E-06 |
| DSC3 | Desmocollin-3 | -1.00 | 6.22E-06 |
| CDR1 | Cerebellar degeneration-related antigen 1 | -1.00 | 1.01E-06 |
| CTGF | Connective tissue growth factor | -1.00 | 6.22E-08 |
